# A unique death pathway keeps RIPK1 D325A mutant mice in check at embryonic day 10.5

**DOI:** 10.1371/journal.pbio.3001304

**Published:** 2021-08-26

**Authors:** Yingying Zhang, Kai Huang, Yuxia Zhang, Tao Han, Lang Li, Chenchen Ruan, Ye-hsuan Sun, Wenke Shi, Wei Han, Su-qin Wu, Jing Song, Jun Liu, Jiahuai Han

**Affiliations:** 1 State Key Laboratory of Cellular Stress Biology, Innovation Center for Cell Biology, School of Life Sciences, Xiamen University, Xiamen, Fujian, China; 2 Laboratory Animal Center, Xiamen University, Xiamen, Fujian, China; 3 Research Unit of Cellular Stress of CAMS, Cancer Research Center of Xiamen University, Xiang’an Hospital of Xiamen University, School of Medicine, Xiamen University, Xiamen, Fujian, China; Universiteit Gent, BELGIUM

## Abstract

Tumor necrosis factor receptor-1 (TNFR1) signaling, apart from its pleiotropic functions in inflammation, plays a role in embryogenesis as deficiency of varieties of its downstream molecules leads to embryonic lethality in mice. Caspase-8 noncleavable receptor interacting serine/threonine kinase 1 (RIPK1) mutations occur naturally in humans, and the corresponding D325A mutation in murine RIPK1 leads to death at early midgestation. It is known that both the demise of *Ripk1*^*D325A/D325A*^ embryos and the death of *Casp8*^*−/−*^ mice are initiated by TNFR1, but they are mediated by apoptosis and necroptosis, respectively. Here, we show that the defects in *Ripk1*^*D325A/D325A*^ embryos occur at embryonic day 10.5 (E10.5), earlier than that caused by *Casp8* knockout. By analyzing a series of genetically mutated mice, we elucidated a mechanism that leads to the lethality of *Ripk1*^*D325A/D325A*^ embryos and compared it with that underlies *Casp8* deletion-mediated lethality. We revealed that the apoptosis in *Ripk1*^*D325A/D325A*^ embryos requires a scaffold function of RIPK3 and enzymatically active caspase-8. Unexpectedly, caspase-1 and caspase-11 are downstream of activated caspase-8, and concurrent depletion of *Casp1* and *Casp11* postpones the E10.5 lethality to embryonic day 13.5 (E13.5). Moreover, caspase-3 is an executioner of apoptosis at E10.5 in *Ripk1*^*D325A/D325A*^ mice as its deletion extends life of *Ripk1*^*D325A/D325A*^ mice to embryonic day 11.5 (E11.5). Hence, an unexpected death pathway of TNFR1 controls RIPK1 D325A mutation-induced lethality at E10.5.

## Abstract 2

A study of mice expressing a caspase-8 non-cleavable RIPK1 mutant during embryonic development reveals an unexpected TNFR1-triggered death pathway involving RIPK3, caspase-8, and caspases -1, -11 and -3.

## Introduction

Tumor necrosis factor (TNF) is a pleiotropic cytokine, which, via its receptor TNF receptor-1 (TNFR1), triggers a variety of cellular responses. Its best-studied function in vivo is mediation of inflammatory diseases such as rheumatoid arthritis, inflammatory bowel disease (Crohn disease and ulcerative colitis), and psoriasis. Actually, TNF–TNFR1 signaling also plays a role in embryonic development. Although neither TNF nor TNFR1 is required for embryonic development in mice [1,2], TNFR1 signaling could eliminate defective embryos at different developmental stages. *Tnfr1* knockout rescues or delays developmental defects caused by deficiency of its downstream effectors such as receptor interacting serine/threonine kinase 1 (RIPK1), caspase-8, CASP8 and FADD-like apoptosis regulator (cFLIP), FAS-associated death domain protein (FADD), ring finger protein 31 (HOIP), RanBP-type and C3HC4-type zinc finger-containing protein 1 (HOIL-1), TANK-binding kinase 1 (TBK1), RelA/p65, cellular inhibitor of apoptosis protein 1 and 2 (cIAP1 and 2), and inhibitor of nuclear factor kappa-B kinase subunit beta (IKKβ) [3–12]. During development at around embryonic day 10.5 (E10.5), mice seem to become sensitive to perturbation of TNFR1 signaling, and lethality at this stage of development has been observed in many mouse models with genetic defects in this pathway such as *Tak1*^*−/−*^, *cIap1*^*−/−*^ *cIap2*^*−/−*^, *Hoip*^*−/−*^, and *Hoil-1*^*−/−*^ [6,10,11,13,14]. The level of involvement of TNFR1 signaling at different developmental stages may differ. For example, deletion of *Tnfr1* rescues embryonic lethality of *Ripk1*^*D325A/D325A*^ mice and allows the mice to survive to around postnatal day 10 (P10), whereas *Tnfr1*^*−/−*^ only delayed the lethality of *Hoip*^*−/−*^ or *Hoil-1*^*−/−*^ embryos to embryonic day 16.5 (E16.5) [6,11,15].

RIPK1 is a protein kinase that plays a key role in regulating TNFR1 signaling pathways. Its recruitment to TNFR1 and ubiquitination allow for assembly of a mitogen-activated protein kinase/nuclear factor-kappa B (MAPK/NF-κB)-activating complex. On the other hand, kinase active RIPK1 can autophosphorylate and interact with RIPK3 and FADD. FADD links RIPK1 to caspase-8, the activation of which can initiate a caspase cascade. Downstream executioner caspases such as caspase-3 are the key effectors of the apoptotic cell death pathway. If, however, caspase-8 activity is inhibited and cellular RIPK3 amount is sufficient, RIPK1 recruits RIPK3 via its RIP homotypic interaction motif (RHIM), thus promoting activation of RIPK3, which, in turn, recruits and phosphorylates mixed lineage kinase domain-like (MLKL). Phosphorylated MLKL then oligomerizes and translocates to the plasma membrane, causing plasma membrane rupture and necroptotic cell death [16,17], which might lead to necroinflammation in vivo [18–20]. Caspase-8 is an aspartate-specific cysteine protease, which can either exert function via its catalytic activity or as a scaffold for complex assembly and signaling transduction. *Casp8*^*−/−*^ mice die at midgestation [21]. Mice carrying catalytically inactive caspase-8 (C362A or C362S mutation) also exhibit embryonic lethality reminiscent of *Casp8*^*−/−*^ mice [15,22]. Caspase-8 catalytic activity is therefore essential for normal embryogenesis, and the underlying mechanism is its inhibition of RIPK1–RIPK3-mediated necroptosis [5,15,22–27]. RIPK1 is a substrate of caspase-8, and noncleavable mutations of RIPK1 occur naturally in humans. Single allele of such mutation causes an early-onset periodic fever syndrome and severe intermittent lymphadenopathy [28,29]. The cleavage site in murine RIPK1 locates at residue D325 [30], and *Ripk1*^*D325A/D325A*^ mice die at an early midgestation day [15,28,31]. Perhaps due to differences in laboratory settings, the lethality was reported to take place at E10.5, E10.5 to 11.5, and embryonic day 12.5 (E12.5), respectively, by different groups [15,28,31]. The death of *Ripk1*^*D325A/D325A*^ embryos at this early midgestation day can be prevented by loss of *Tnfr1*, RIPK1 kinase activity, or *Ripk3*, although the double mutant mice still died later on [15,28,31], seemingly suggesting that the defects of *Ripk1*^*D325A/D325A*^ mice at early midgestation are due to not being able to be cleaved by caspase-8 and that caspase-8 cleavage of RIPK1 inhibits TNFR1–RIPK3-mediated RIPK1 kinase activity-dependent cell death at early midgestation of embryonic development.

Caspase-1 and caspase-11 are inflammatory caspases well studied for their roles in inflammasome activation and are required for IL-1β and IL-18 processing and maturation and for pyroptosis in response to pathogen patterns and endogenous danger stimuli. While there is a large amount of evidence obtained by using genetic knockout mice showing that caspase-1 and/or caspase-11 are indispensable in host responses against certain microbial pathogens [32–35] and in driving sterile inflammation [36–38], mice deficient in *Casp1*, *Casp11*, or both *Casp1* and *Casp11* are developmentally normal, and knowledge on their functions in other biological processes besides inflammation is limited. Their functions in embryonic development are unknown.

Here, with genetic evidence, we show that the effect of caspase-8 noncleavable RIPK1 mutation does not mimic that of *Casp8* deletion. This mutation arouses death signaling at E10.5, which is earlier than that caused by *Casp8* deletion at embryonic day 11.5 (E11.5). TNFR1 is required for the initiation of both death processes, but RIPK1(D325A)-mediated E10.5 lethality is resulted from RIPK3- and caspase-8–dependent apoptosis, whereas *Casp8* knockout-mediated embryonic death is caused by necroptosis. Furthermore, caspase-1 and caspase-11 are found, for the first time to our knowledge, to function during embryogenesis, downstream of caspase-8, leading to caspase-3-dependent apoptosis at E10.5. The death pathway activated by RIPK1 D325A at E10.5 comprises signaling molecules that are known to participate in necroptosis, extrinsic apoptosis, or pyroptosis, making it unique compared to the known signaling pathways of cell death. TNFR1 thus could initiate different death pathways at different embryonic days to eliminate defective embryos caused by distinct genetic errors.

## Results

### The embryonic lethality caused by D325A mutation in *Ripk1* occurs earlier than that triggered by *Casp8* knockout in mice

**Fig 1:**
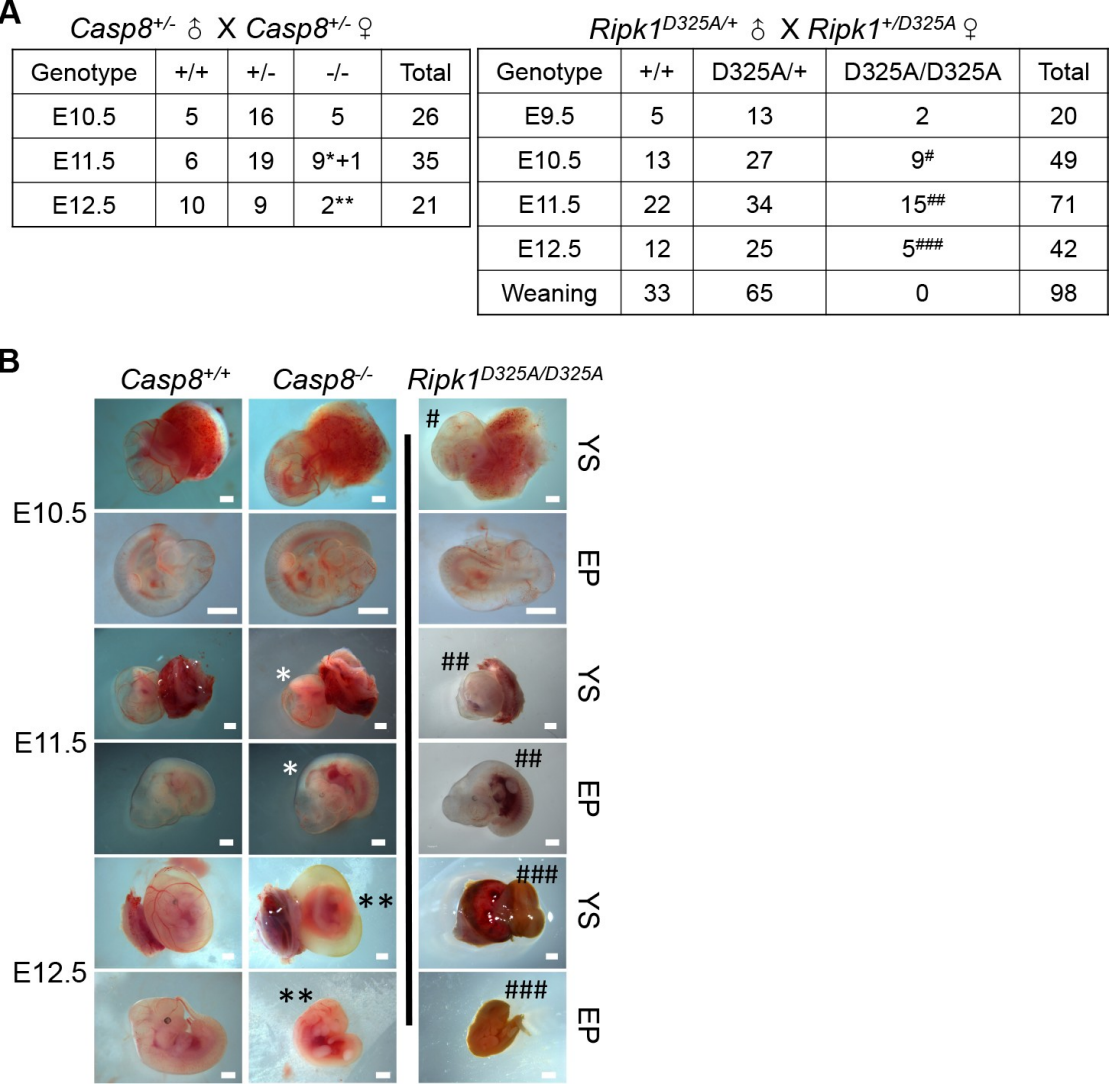
The embryonic lethality caused by D325A mutation in *Ripk1* occurs earlier than that triggered by *Casp8* knockout in mice. **(A)** Genetic analysis of offspring from intercrosses of *Casp8*^*+/−*^ and *Ripk1*^*D325A/+*^ parents, respectively. *: less and thinner YS vessels and signs of hyperemia in the abdominal area in E11.5 embryos; **: severe YS vascularization defect and dead EP in E12.5 embryos; #: defective vascularization in E10.5 YS and normal EP; ##: no vessels in E11.5 YS and severe abdominal hemorrhage in the EP; ###: dead and resorbed embryos at E12.5. **(B)** Representative images of E10.5, E11.5, and E12.5 embryos described in **(A)**. Scale bars, 1 mm. See also S1 Fig. E10.5, embryonic day 10.5; E11.5, embryonic day 11.5; E12.5, embryonic day 12.5; EP, embryo proper; *Ripk1*, receptor interacting serine/threonine kinase 1; YS, yolk sac.

Published studies showed that the D325A mutation in murine *Ripk1*, which resembles naturally occurring D324 to V, H, N, or Y mutation in humans, resists caspase-8 cleavage and leads to embryonic lethality in mice [15,28,29,31]. An interpretation for this phenomenon is that D325A mutation prevents caspase-8–mediated cleavage of RIPK1 and thus mimics the effect of *Casp8* knockout, i.e., promoting RIPK1–RIPK3 necrosome-mediated necroptosis in mouse embryos [31]. But excessive cleaved caspase-3 observed in *Ripk1*^*D325A/D325A*^ embryos by previous work challenged this interpretation [15,28], indicating an involvement of apoptosis. The observation by previous studies that *Mlkl* deletion fails to rescue the lethality of *Ripk1*^*D325A/D325A*^ mice while concomitant loss of *Mlkl* plus *Fadd* can do further supports the role of apoptosis in the lethality of *Ripk1*^*D325A/D325A*^ mice [15,28]. Because deletion of *Mlkl* did not have any survival benefit on *Ripk1*^*D325A/D325A*^ embryos whereas deletion of *Ripk3* delayed death of *Ripk1*^*D325A/D325A*^ embryos to E16.5 [15,28,31], a necroptosis-independent function of RIPK3 should play a role in E10.5 lethality of *Ripk1*^*D325A/D325A*^ mice [15,28]. We had also generated *Ripk1*^*D325A/D325A*^ and *Casp8*^*−/−*^ mice and analyzed these mice in our laboratory (S1A Fig). Similar to published results [21], *Casp8*^*−/−*^ embryos appeared normal at E10.5, the majority of the embryos showed less and thinner yolk sac vessels and signs of hyperemia in the abdominal area at E11.5, and all of them displayed defective yolk sac vascularization and dead embryo proper at E12.5 (Fig 1A and 1B). Different from *Casp8*^*−/−*^ embryos, the onset of defects in *Ripk1*^*D325A/D325A*^ embryos was significantly earlier, and they were more severe (Fig 1A and 1B). Timed mating analysis revealed that all of *Ripk1*^*D325A/D325A*^ progeny exhibited yolk sac vascularization defects at E10.5, approximately 1 to 2 days earlier than *Casp8*^*−/−*^ embryos, and displayed severe abdominal hemorrhage in the embryo proper, while *Casp8*^*−/−*^ embryos just started to show defects at E11.5. All D325A homozygous embryos were resorbed at E12.5 and could not be detected after this developmental stage (Fig 1B). Furthermore, *Ripk1*^*D325A/−*^ offspring from crosses of *Ripk1*^*D325A/+*^ and *Ripk1*^*+/−*^ parents were normal at E10.5 and died perinatally between embryonic day 17.5 (E17.5) and postnatal day 1 (P1), a phenotype resembling that of *Ripk1*^*−/−*^ mice [5,25,26,39], indicating a dose effect of RIPK1 D325A mutation (S1B–S1D Fig). The above data, together with the published results that *Mlkl* deletion can rescue the lethality of *Casp8*^*−/−*^ mice but not that of *Ripk1*^*D325A/D325A*^ mice [15,28], indicate that the mechanism causing the lethality of *Ripk1*^*D325A/D325A*^ mice is different from that of *Casp8*^*−/−*^ mice.

### Defect of *Ripk1*^*D325A/D325A*^ mice at E10.5 is mediated by TNFR1–RIPK3–caspase-8 signaling axis

Lethality of *Casp8*^*−/−*^ embryos at E11.5 appears to be driven by TNFR1-mediated necroptosis as it can be postponed by individual gene deletion of *Tnfr1* or *Ripk1* and can be fully rescued by loss of *Ripk3* or *Mlkl* [5,23–27]. Similarly, lethality of *Ripk1*^*D325A/D325A*^ mice at E10.5 can be rescued by *Tnfr1*^*−/−*^ [15] to up to P10 (S2A and S2B Fig). But in sharp contrast to *Casp8*^*−/−*^ mice, *Mlkl* deficiency failed to rescue the defects of *Ripk1*^*D325A/D325A*^ embryos [15,28], and deletion of *Ripk3* only delayed the death of *Ripk1*^*D325A/D325A*^ mice to E16.5 [15,28,31] (S2C, S2D, S3B, and S3C Figs), indicating that although the death of both *Casp8*^*−/−*^ and *Ripk1*^*D325A/D325A*^ embryos is triggered by TNFR1, one undergoes RIPK3–MLKL-mediated necroptosis, while the other does not. Indeed, we cannot detect MLKL phosphorylation in *Ripk1*^*D325A/D325A*^ yolk sacs by immunofluorescence (IF) staining (S2E Fig). In addition, published studies already identified cleaved caspase-3 in E10.5 *Ripk1*^*D325A/D325A*^ embryos, indicating an apoptotic phenotype [15,28]. This suggests that the mechanism of TNFR1-mediated lethality of *Ripk1*^*D325A/D325A*^ mice at E10.5 should differ from that of TNFR1-mediated E11.5 lethality of *Casp8*^*−/−*^ mice.

**Fig 2:**
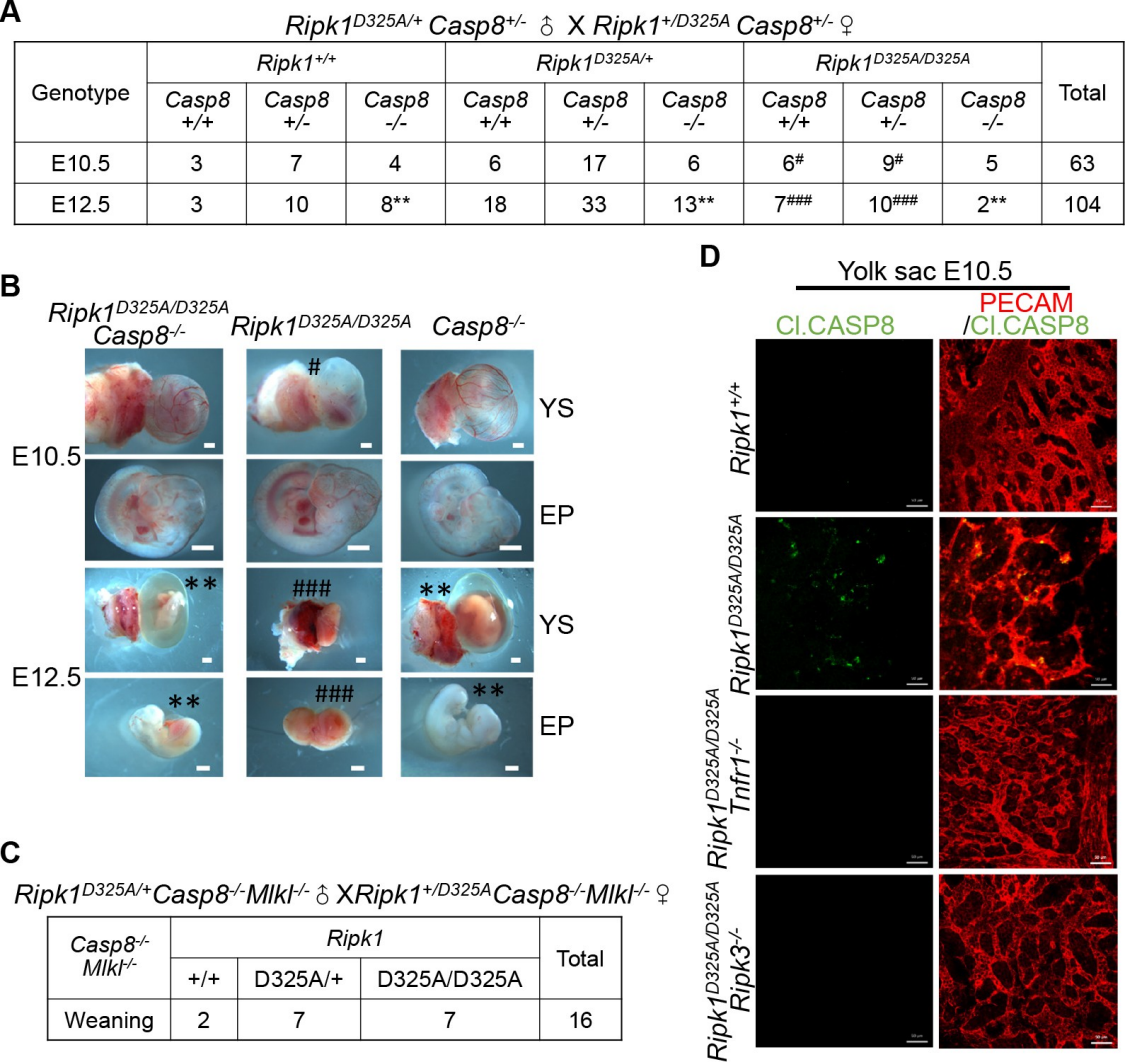
Defect of *Ripk1*^*D325A/D325A*^ mice at E10.5 is mediated by TNFR1–RIPK3–caspase-8 signaling axis. **(A)** Genetic analysis of progeny from intercrossing *Ripk1*^*D325A/+*^ *Casp8*^*+/−*^ mice. **(B)** Representative images of E10.5 and E12.5 embryos analyzed in **(A)**. Scale bars, 1 mm. **(C)** Genetic analysis of offspring from intercrossing *Ripk1*^*D325A/+*^ *Casp8*^*−/−*^ *Mlkl*^*−/−*^ parents. **(D)** IF staining of E10.5 YS with anti-PECAM (red) and anti-Cl.CASP8 (green) antibodies. Scale bars, 50 μm. Images are representative of 4 embryos per genotype. See also S2 and S3 Figs. Cl.CASP8, cleaved caspase-8; E10.5, embryonic day 10.5; E12.5, embryonic day 12.5; EP, embryo proper; IF, immunofluorescence; PECAM, platelet endothelial cell adhesion molecule; *Ripk1*, receptor interacting serine/threonine kinase 1; TNFR1, tumor necrosis factor receptor-1; YS, yolk sac.

Since the E10.5 defect of *Ripk1*^*D325A/D325A*^ mice is not mediated by necroptosis and possibly by apoptosis, we examined *Ripk1*^*D325A/D325A*^ *Casp8*^*−/−*^ embryos and surprisingly found that while the *Ripk1*^*D325A/D325A*^ littermates were defective in the yolk sac, *Ripk1*^*D325A/D325A*^ *Casp8*^*−/−*^ embryos were normal at E10.5, indicating the involvement of caspase-8 in the E10.5 lethality (Fig 2A and 2B). As expected, none of the *Ripk1*^*D325A/D325A*^ *Casp8*^*−/−*^ embryos were alive when they were analyzed at E12.5 (Fig 2A and 2B). Thus, the death of *Ripk1*^*D325A/D325A*^ *Casp8*^*−/−*^ embryos is likely due to the deficiency of *Casp8*. Indeed, the lethality of *Ripk1*^*D325A/D325A*^ *Casp8*^*−/−*^ embryos can be rescued by *Mlkl*^*−/−*^ to adulthood, similar to *Casp8*^*−/−*^ embryos (Fig 2C), and the resulting *Ripk1*^*D325A/D325A*^ *Casp8*^*−/−*^ *Mlkl*^*−/−*^ mice exhibited similar *lpr* phenotypes (lymphadenopathy and splenomegaly) to those of *Casp8*^*−/−*^ *Mlkl*^*−/−*^ mice [27,40] (S3A Fig). Moreover, caspase-8 cleavage was observed in E10.5 *Ripk1*^*D325A/D325A*^ yolk sacs by IF staining (Fig 2D). Collectively, our data indicated that E10.5 lethality of *Ripk1*^*D325A/D325A*^ embryos requires caspase-8.

The rescue of *Ripk1*^*D325A/D325A*^ embryos by *Ripk3* deletion to either E12.5 or E16.5 was reported independently [28,31]. We could obtain normal E16.5 embryos with *Ripk1*^*D325A/D325A*^*Ripk3*^*−/−*^ genotype (S3B and S3C Fig). This rescue is unlikely resulted from blockade of necroptosis because loss of *Mlkl* had no effect on the lethality of *Ripk1*^*D325A/D325A*^ embryos [15,28] (S2C and S2D Fig). Thus, the E10.5 lethality should be mediated by an MLKL-independent function of RIPK3. Since caspase-8 activation was revealed in *Ripk1*^*D325A/D325A*^ yolk sacs but cannot be detected in both *Ripk1*^*D325A/D325A*^ *Tnfr1*^*−/−*^ and *Ripk1*^*D325A/D325A*^ *Ripk3*^*−/−*^ yolk sacs (Fig 2D), caspase-8 activation in *Ripk1*^*D325A/D325A*^ embryos should be downstream of TNFR1 and RIPK3. Thus, the embryonic death of *Ripk1*^*D325A/D325A*^ mice is triggered by a signaling from TNFR1 to RIPK3 and then caspase-8.

### Enzymatic activity of caspase-8 and RIPK1 but not RIPK3 is required for E10.5 lethality of *Ripk1*^*D325A/D325A*^ mice

**Fig 3:**
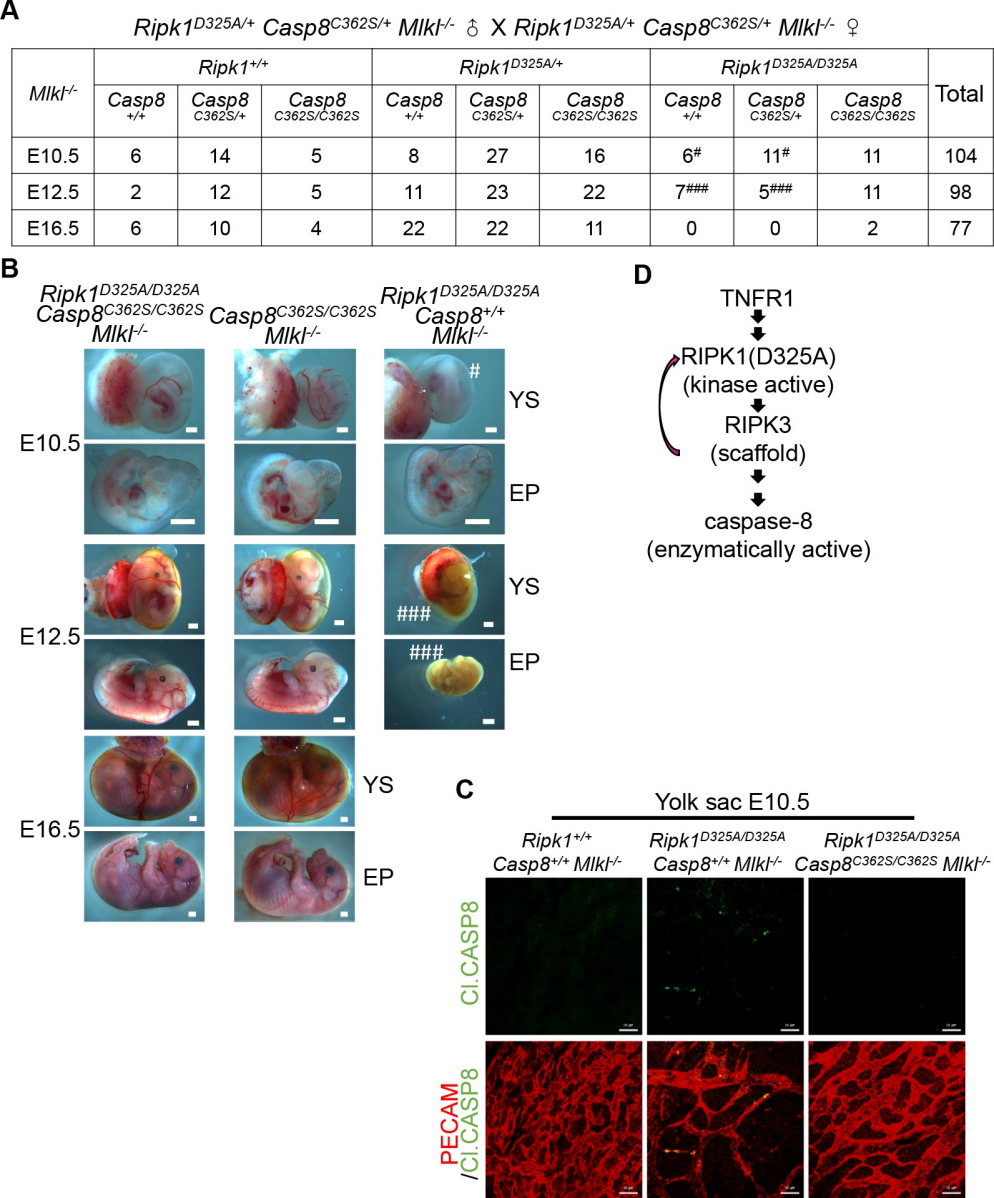
Enzymatic activity of caspase-8 and RIPK1 but not RIPK3 is required for E10.5 lethality of *Ripk1*^*D325A/D325A*^ mice. **(A)** Genetic analysis of progeny from intercrossing *Ripk1*^*D325A/+*^ *Casp8*^*C362S/+*^ *Mlkl*^*−/−*^ parents. **(B)** Representative E10.5, E12.5, and E16.5 embryos collected in **(A)**. Scale bars, 1 mm. **(C)** IF staining of E10.5 YS with anti-PECAM (red) and anti-Cl.CASP8 (green) antibodies. Scale bars, 50 μm. Images are representative of 4 embryos per genotype. **(D)** Schematic diagram of the signaling transduction elucidated. See also S4 Fig. Cl.CASP8, cleaved caspase-8; E10.5, embryonic day 10.5; E12.5, embryonic day 12.5; E16.5, embryonic day 16.5; EP, embryo proper; IF, immunofluorescence; PECAM, platelet endothelial cell adhesion molecule; RIPK1, receptor interacting serine/threonine kinase 1; TNFR1, tumor necrosis factor receptor-1; YS, yolk sac.

Caspase-8 could either exert function via its catalytic activity or as a scaffold. To dissect its function of scaffold and protease activity, we generated homozygous *Casp8*^*C362S/C362S*^ mice in which caspase-8 catalytic activity is completely lost. As reported earlier, *Casp8*^*C362S/C362S*^ embryos phenocopied *Casp8*^*−/−*^ embryos, which started to show defects at E11.5 and were all dead at E12.5 (S4A and S4B Fig), and *Mlkl*^*−/−*^postponed the death of *Casp8*^*C362S/C362S*^ embryos to birth [15,22]. We crossed *Ripk1*^*D325A/+*^ mice onto the *Casp8*^*C362S/+*^ *Mlkl*^*−/−*^ background and surprisingly found that the resulting *Ripk1*^*D325A/D325A*^ *Casp8*^*C362S/C362S*^ *Mlkl*^*−/−*^ embryos were normal at E10.5, E12.5, and E16.5 (Fig 3A and 3B), demonstrating that catalytic activity of caspase-8 is required for the E10.5 lethality of *Ripk1*^*D325A/D325A*^ mice. In addition to this genetic evidence, caspase-8 activation in E10.5 *Ripk1*^*D325A/D325A*^ and *Ripk1*^*D325A/D325A*^ *Mlkl*^*−/−*^ yolk sacs was revealed by IF staining of cleaved caspase-8, but not in *Ripk1*^*D325A/D325A*^ *Casp8*^*C362S/C362S*^ *Mlkl*^*−/−*^ yolk sacs (Figs 2D and 3C), suggesting that caspase-8 activation is mediated by its auto-processing.

It is well known that RIPK1 can function as a kinase and/or a scaffold. Additional kinase inactivation mutation (*Ripk1*^*Δ/Δ*^) [41] of *Ripk1* rescued embryonic death of *Ripk1*^*D325A/D325A*^ mice to P8 (S4C Fig). Thus, as concluded by others [15,28], RIPK1 kinase activity is required for E10.5 lethality of *Ripk1*^*D325A/D325A*^ mice.

RIPK3 likewise plays kinase and scaffold roles. RIPK3 activation is required for necroptosis and auto-phosphorylation on T231 and S232 of murine RIPK3 is a signature of RIPK3 activation [42]. We analyzed E10.5 *Ripk1*^*D325A/D325A*^ yolk sacs by IF staining and found no RIPK3 auto-phosphorylation (S4D Fig), indicating that RIPK3 activation is not required for the E10.5 defects of *Ripk1*^*D325A/D325A*^ mice. Since RIPK3 cannot interact with caspase-8 directly but can function as a scaffold to activate caspase-8 via interaction with RIPK1 in the presence of a RIPK3 inhibitor GSK-872 [43], RIPK3 here is most likely a scaffold of RIPK1. The idea that it is the scaffold rather than kinase activity of RIPK3 that determines the death of *Ripk1*^*D325A/D325A*^ embryos has been proposed [15,28]. Our data support this notion (S2E and S4D Figs), although D325A mutation is known to promote RIPK3 activation and necroptosis in some cell-based systems and perhaps in other biological processes [15,28,44]. In summary, the E10.5 lethality of *Ripk1*^*D325A/D325A*^ mice requires caspase-8 activity, RIPK1 kinase activity, and RIPK3 scaffold function (Fig 3D).

### Concurrent deficiency of *Casp1* and *Casp11* is required for prevention of E10.5 lethality of *Ripk1*^*D325A/D325A*^ mice

**Fig 4:**
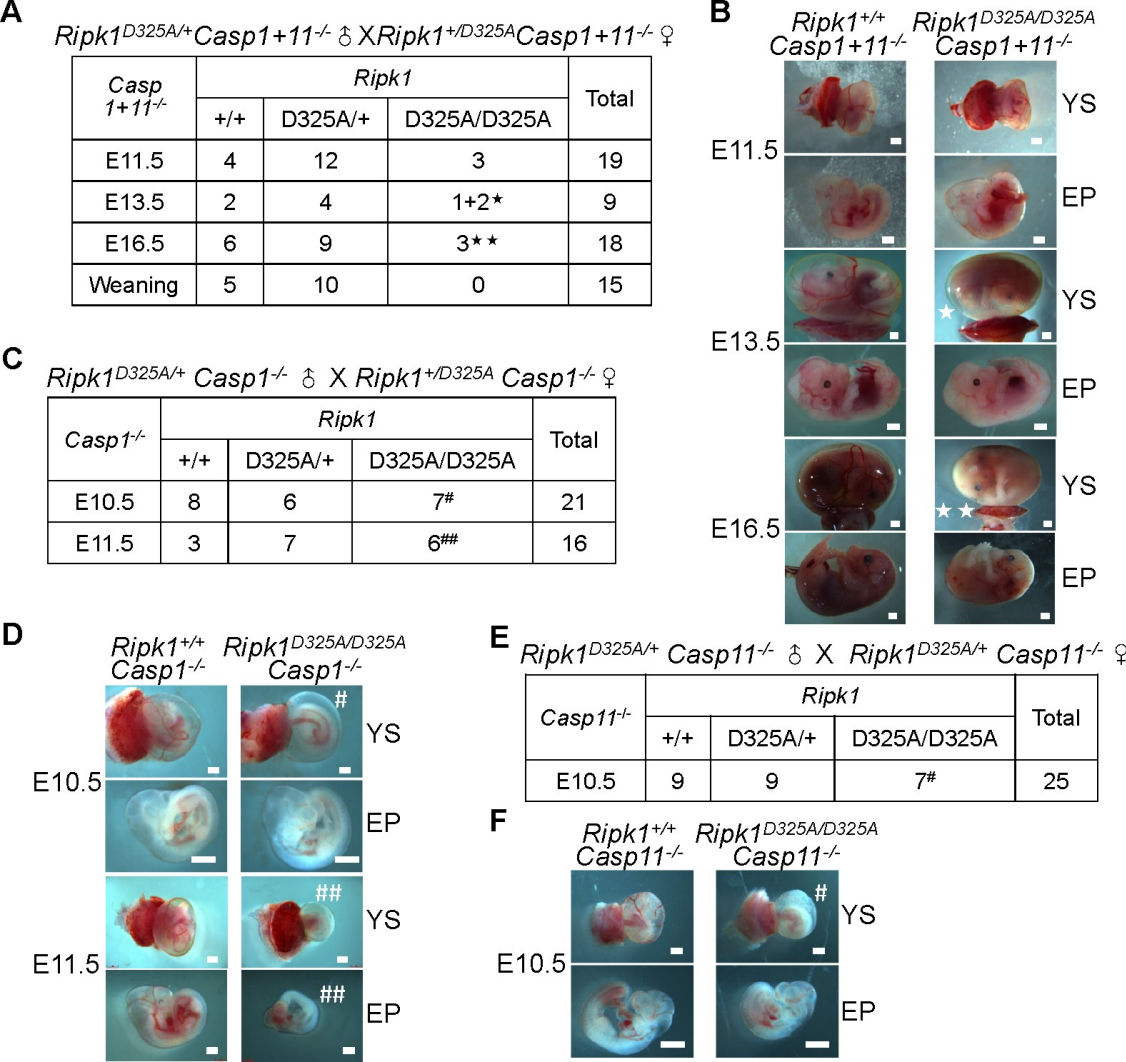
Concurrent deficiency of *Casp1* and *Casp11* is required for prevention of E10.5 lethality of *Ripk1*^*D325A/D325A*^ mice. **(A)** Genetic analysis of offspring from intercrosses of *Ripk1*^*D325A/+*^ *Casp1+11*^*−/−*^ parents. ★: YS vascularization defect and normal EP. ★★: YS vascularization defect and smaller and paler EP. **(B)** Representative E11.5, E13.5, and E16.5 embryos obtained in **(A)**. Scale bars, 1 mm. **(C)** Genetic analysis of progeny from intercrossing *Ripk1*^*D325A/+*^ *Casp1*^*−/−*^ parents. **(D)** Representative E10.5 and E11.5 embryos collected in **(C)**. Scale bars, 1 mm. **(E)** Genetic analysis of progeny from intercrosses of *Ripk1*^*D325A/+*^ *Casp11*^*−/−*^ mice. **(F)** Representative E10.5 embryos analyzed in **(E)**. Scale bars, 1 mm. See also S5A Fig. E10.5, embryonic day 10.5; E11.5, embryonic day 11.5; E13.5, embryonic day 13.5; E16.5, embryonic day 16.5; EP, embryo proper; *Ripk1*, receptor interacting serine/threonine kinase 1; YS, yolk sac.

Recent publications suggest that there is crosstalk and sharing of signaling components among death pathways, such as caspase-8 and inflammasome components [45–56]. We were therefore curious whether inflammasome components also participate in *Ripk1*^*D325A/D325A*^ lethality. Astonishingly, combined loss of *Casp1* and *Casp11* (*Casp1+11*^*−/−*^) rescued E10.5 defects of *Ripk1*^*D325A/D325A*^ mice, although the mice still died later during embryogenesis (Figs 4A, 4B, and S5A). *Ripk1*^*D325A/D325A*^ *Casp1+11*^*−/−*^ embryos were normal at E11.5, started to display fewer blood vessels in the yolk sac at E13.5, and were all dead by E16.5 (Fig 4A and 4B). We then generated *Casp1*^*−/−*^ mice and *Casp11*^*−/−*^ mice (S5A Fig), respectively, to address the roles of caspase-1 and caspase-11. Timed mating analysis showed that neither *Casp1*^*−/−*^ nor *Casp11*^*−/−*^ can rescue the E10.5 defects of *Ripk1*^*D325A/D325A*^ mice (Fig 4C–4F). Therefore, concomitant deletion of *Casp1* and *Casp11* is required for blocking E10.5 lethality of *Ripk1*^*D325A/D325A*^ mice.

### Caspase-8 is upstream of caspase-1 in the death pathway of *Ripk1*^*D325A/D325A*^ mice at E10.5

**Fig 5:**
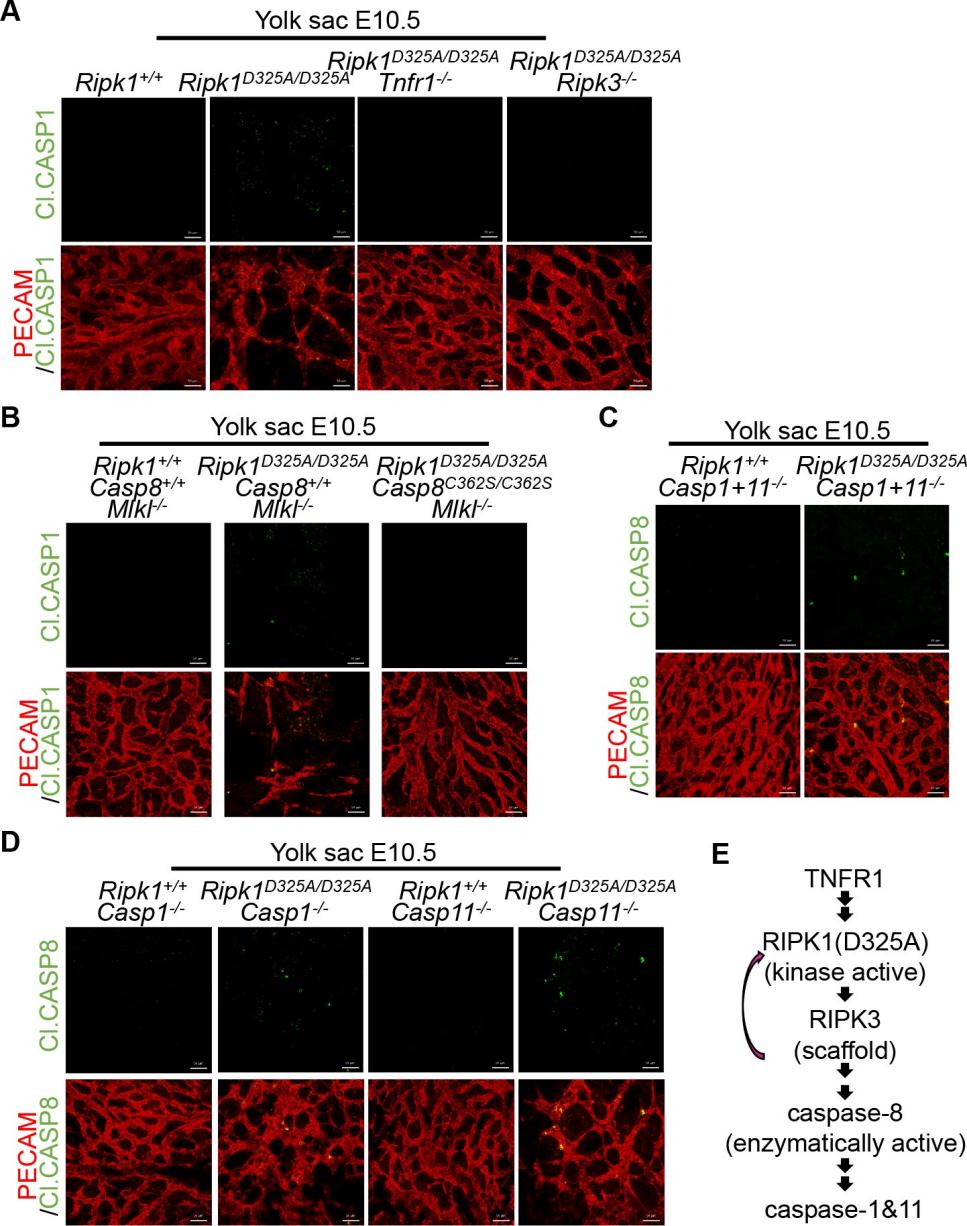
Caspase-8 is upstream of caspase-1 in the death pathway of *Ripk1*^*D325A/D325A*^ mice at E10.5. **(A and B)** IF staining of E10.5 YS with anti-PECAM (red) and anti-Cl.CASP1 (green) antibodies. Scale bars, 50 μm. Images are representative of 4 embryos per genotype. **(C and D)** IF staining of E10.5 YS with anti-PECAM (red) and anti-Cl.CASP8 (green) antibodies. Scale bars, 50 μm. Images are representative of 4 embryos per genotype. **(E)** Schematic diagram of the signaling transduction. See also S5B–S5G Fig. Cl.CASP1, cleaved caspase-1; Cl.CASP8, cleaved caspase-8; E10.5, embryonic day 10.5; IF, immunofluorescence; PECAM, platelet endothelial cell adhesion molecule; RIPK1, receptor interacting serine/threonine kinase 1; TNFR1, tumor necrosis factor receptor-1; YS, yolk sac.

We then sought to address the relationship between caspase-1, caspase-11, and the other known signaling components in the E10.5 lethality of *Ripk1*^*D325A/D325A*^ mice. Since there is no suitable antibody to detect the activation of caspase-11, we only analyzed caspase-1 cleavage in E10.5 yolk sacs of wild-type (WT), *Ripk1*^*D325A/D325A*^, *Ripk1*^*D325A/D325A*^ *Tnfr1*^*−/−*^, *Ripk1*^*D325A/D325A*^ *Ripk3*^*−/−*^, and *Ripk1*^*D325A/D325A*^ *Casp8*^*C362S/C362S*^ *Mlkl*^*−/−*^ embryos (Fig 5A and 5B). The yolk sacs of E10.5 *Ripk1*^*D325A/D325A*^ embryos and *Ripk1*^*D325A/D325A*^ *Mlkl*^*−/−*^ embryos were stained positive for cleaved caspase-1, while there was no signal in the WT or *Mlkl*^*−/−*^ control (Fig 5A and 5B). Genetic deletion of *Tnfr1* or *Ripk3*, or C362S mutation of *Casp8* all blocked caspase-1 activation (Fig 5A and 5B), whereas caspase-8 activation was easily detected in E10.5 yolk sacs of *Ripk1*^*D325A/D325A*^ *Casp1+11*^*−/−*^, *Ripk1*^*D325A/D325A*^ *Casp1*^*−/−*^, and *Ripk1*^*D325A/D325A*^ *Casp11*^*−/−*^ embryos (Fig 5C and 5D), indicating that caspase-1 is downstream of caspase-8. Since deficiencies of *Casp1* and *Casp11* are simultaneously required for prevention of E10.5 lethality of *Ripk1*^*D325A/D325A*^ mice, caspase-1 and caspase-11 should function in parallel rather than up or downstream of each other. Provided the fact that caspase-1 is downstream of caspase-8, caspase-11 is likely to be the same. Therefore, we proposed that caspase-1 and caspase-11 were activated in parallel at the downstream of caspase-8 activation in E10.5 *Ripk1*^*D325A/D325A*^ embryos (Fig 5E).

The capability of caspase-8 to cleave caspase-1 and caspase-11 directly has been documented by in vitro experiments [57]. Due to the technical difficulty in obtaining a sufficient number of endothelial cells from the yolk sacs, we are unable to determine whether caspase-8 can directly activate caspase-1/11 in the yolk sacs. We isolated mouse embryonic fibroblasts (MEFs) and tried to address this issue using TNF-treated MEFs. As previously reported [15,28,31], TNF treatment led to a small amount of cell death in WT MEFs and much more death in *Ripk1*^*D325A/D325A*^ MEFs (S5B Fig). Additional deletion of *Casp1+11* did not bring any extra effect on TNF-induced cell death on a *Ripk1*^*D325A/D325A*^ background (S5B Fig). We analyzed the expression level of proteins related to cell death in these TNF-treated MEFs by western blot and did not detect the expression of caspase-1 or caspase-11 in MEFs; thus, the involvement of caspase-1 and caspase-11 cannot be studied using MEFs (S5C Fig). We performed immunoprecipitation of TNFR1 complex and found increased recruitment of RIPK1 especially shifted RIPK1 in the receptor super complex when D325 was mutated to A (S5D Fig). The recruitment of TNFR1-associated death domain protein (TRADD), A20, and linear ubiquitin chain assembly complex (LUBAC) component SHANK-associated RH domain interacting protein (SHARPIN) was also increased in *Ripk1*^*D325A/D325A*^ MEFs. The increase of TNFR1 complex formation may explain at least in part the increased sensitivity of D325A mutant MEFs to TNF-induced cell death. We then performed immunoprecipitation of RIPK1 to evaluate complex II formation. We did not detect coprecipitated FADD, caspase-8, or RIPK3 in TNF− or TNF + SMAC mimetic (TS)-treated WT and *Ripk1*^*D325A/D325A*^ MEFs but detected FADD, caspase-8, and RIPK3 in the RIPK1 immunoprecipitates in TNF + SMAC mimetic + zVAD (TSZ)-treated cells (S5E–S5G Fig). D325A mutation did not enhance the formation of necrosome, i.e., RIPK1 immunoprecipitates in TSZ-treated MEFs (S5G Fig). It is possible that the formation of complex II was transient even in TS-treated MEFs, and only zVAD-preserved necrosome was detected in our experiments.

### Neither intrinsic apoptosis nor pyroptosis is required for *Ripk1*^*D325A/D325A*^ lethality

**Fig 6:**
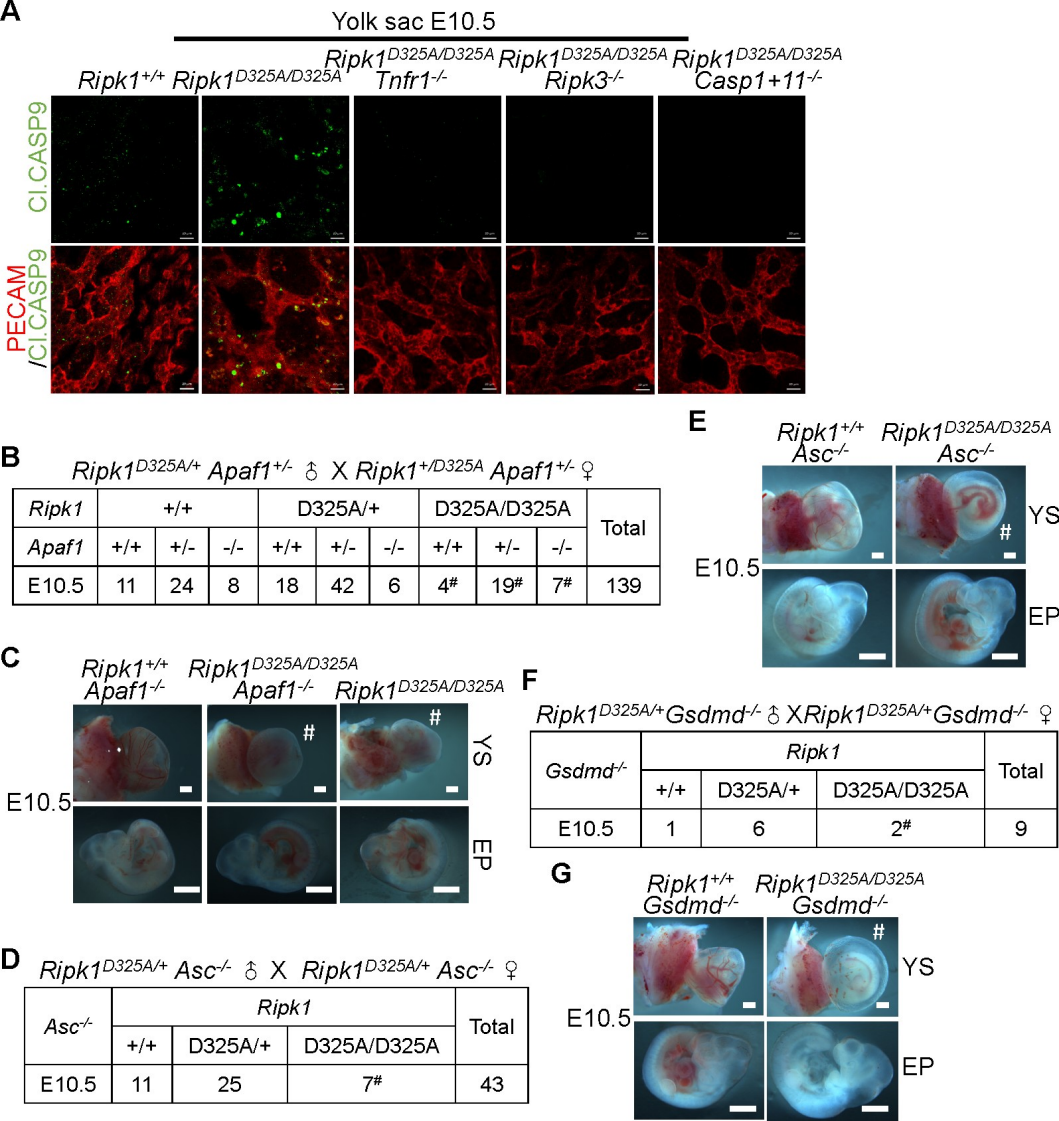
Neither intrinsic apoptosis nor pyroptosis is required for *Ripk1*^*D325A/D325A*^ lethality. **(A)** IF staining of E10.5 YS with anti-PECAM (red) and anti-Cl.CASP9 (green) antibodies. Scale bars, 50 μm. Images are representative of 4 embryos per genotype. **(B)** Genetic analysis of progeny from intercrossing *Ripk1*^*D325A/+*^ *Apaf1*^*+/−*^ parents. **(C)** Representative E10.5 embryos collected in **(B)**. Scale bars, 1 mm. **(D)** Genetic analysis of offspring from intercrossing *Ripk1*^*D325A/+*^ *Asc*^*−/−*^ parents. **(E)** Representative E10.5 embryos obtained in **(D)**. Scale bars, 1 mm. **(F)** Genetic analysis of offspring from intercrosses of *Ripk1*^*D325A/+*^ *Gsdmd*^*−/−*^ mice. **(G)** Representative E10.5 embryos collected in **(F)**. Scale bars, 1 mm. See also S6A–S6C Fig. Cl.CASP9, cleaved caspase-9; E10.5, embryonic day 10.5; EP, embryo proper; IF, immunofluorescence; PECAM, platelet endothelial cell adhesion molecule; *Ripk1*, receptor interacting serine/threonine kinase 1; YS, yolk sac.

We then set out to seek for events downstream of caspase-1 and caspase-11. To our surprise, IF staining revealed caspase-9 cleavage in *Ripk1*^*D325A/D325A*^ yolk sacs but not in *Ripk1*^*D325A/D325A*^ *Tnfr1*^*−/−*^, *Ripk1*^*D325A/D325A*^ *Ripk3*^*−/−*^, or *Ripk1*^*D325A/D325A*^ *Casp1+11*^*−/−*^ yolk sacs, suggesting a possible role of intrinsic apoptotic signaling in the death of *Ripk1*^*D325A/D325A*^ mice (Fig 6A). Apoptotic protease activating factor 1 (Apaf-1) is the core component of apoptosome in the intrinsic pathway, the formation of which leads to caspase-9 activation and the subsequent caspase cascade. The majority of *Apaf1*^*−/−*^ mice die perinatally, and only 5% of the homozygotes survive this period [58]. To find out the contribution of intrinsic apoptosis in *Ripk1*^*D325A/D325A*^ lethality, *Ripk1*^*D325A/D325A*^ *Apaf1*^*−/−*^ mice were generated (S6A Fig). Surprisingly, although cleaved caspase-9 was detected in *Ripk1*^*D325A/D325A*^ embryos, loss of *Apaf1* was unable to rescue the E10.5 defects of *Ripk1*^*D325A/D325A*^ mice (Fig 6B and 6C). Therefore, activation of the intrinsic apoptotic pathway is not required for the E10.5 lethality of *Ripk1*^*D325A/D325A*^ mice.

Since caspase-1 and caspase-11 can activate GSDMD-mediated pyroptosis and the inflammasome adaptor apoptosis-associated speck-like protein containing a CARD (ASC) was reported to facilitate caspase-8–caspase-1 signaling [45,59], and caspase-8 can also cleave GSDMD under certain conditions [60–63], we wondered whether pyroptosis occurs in *Ripk1*^*D325A/D325A*^ embryos. We crossed *Ripk1*^*D325A/+*^ mice onto the *Asc*^*−/−*^ or *Gsdmd*^*−/−*^ background and found the deletion of neither *Asc* nor *Gsdmd* rescued E10.5 lethality of *Ripk1*^*D325A/D325A*^ mice (Fig 6D–6G). Accordingly, no ASC or GSDMD activation in E10.5 *Ripk1*^*D325A/D325A*^ yolk sacs was detected (S6B Fig). We analyzed expression of the gasdermin family members in yolk sacs and did not detect the expression of GSDMD, GSDMA, GSDMC, or GSDME (DFNB59) (S6C Fig), suggesting that none of them is likely to play a role in E10.5 lethality of *Ripk1*^*D325A/D325A*^ embryos. Taken together, these results suggest that neither the intrinsic apoptotic nor the pyroptotic pathway is required for E10.5 lethality of *Ripk1*^*D325A/D325A*^ mice.

### Caspase-3 participates in the E10.5 lethality of RIPK1 D325A mutant mice

**Fig 7:**
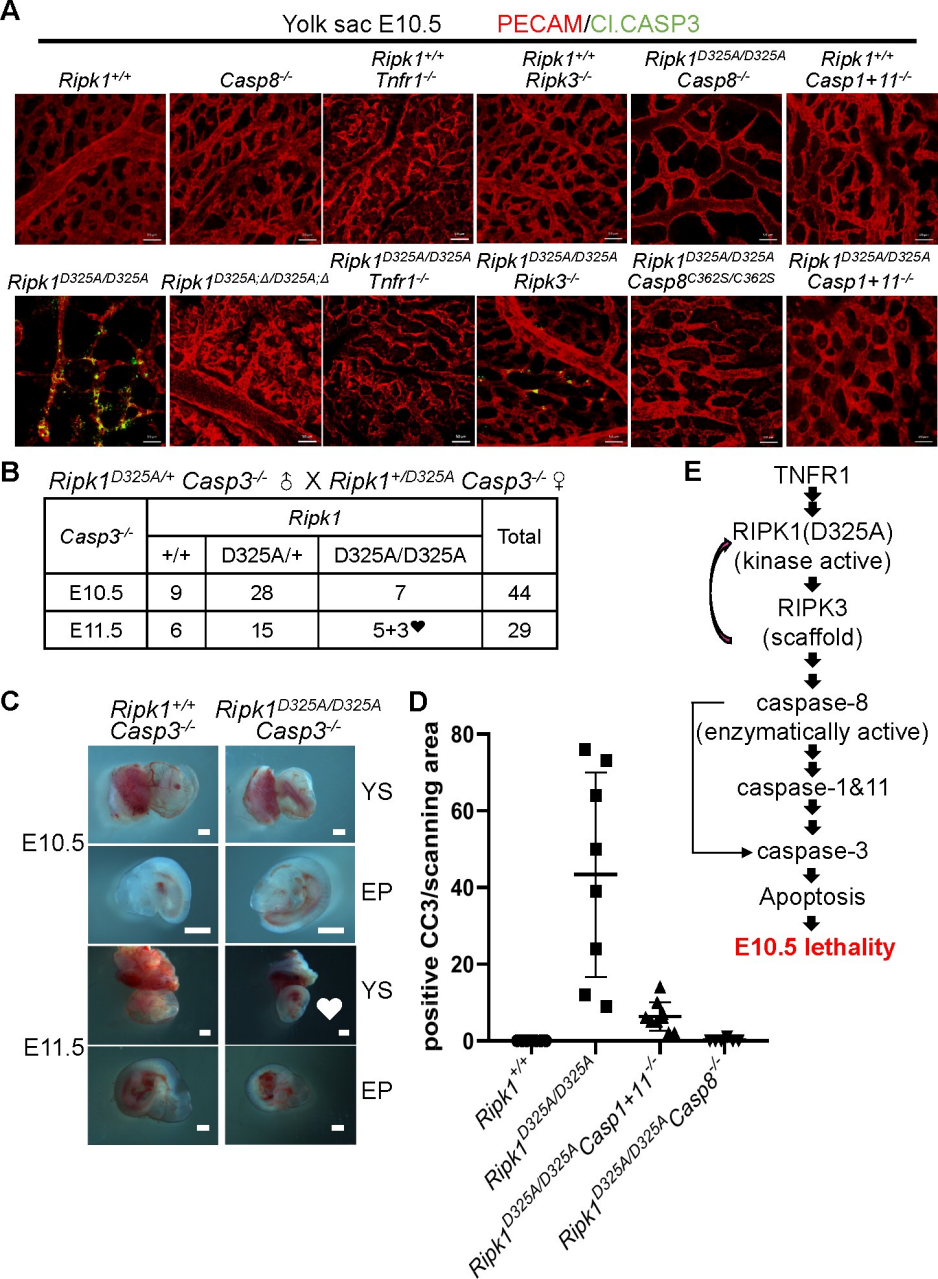
Caspase-3 participates in the E10.5 lethality of RIPK1 D325A mutant mice. **(A)** IF staining of E10.5 YS of indicated genotypes with anti-PECAM (red) and anti-Cl.CASP3 (green) antibodies. Scale bars, 50 μm. Images are representative of 4 embryos per genotype. **(B)** Genetic analysis of offspring from intercrosses of *Ripk1*^*D325A/+*^ *Casp3*^*−/−*^ parents. **♥:** YS vascularization defects and smaller EP with hyperaemia. **(C)** Representative E10.5 and E11.5 embryos obtained in **(B)**. Scale bars, 1 mm. **(D)** Quantitative analysis of YS cells positive for Cl.CASP3 immunostaining per scanning area. At least 6 areas were analyzed for each genotype. **(E)** Schematic diagram of the signaling pathway deciphered. Underlying data are available in S1 Data. See also S6D–S6F Fig. Cl.CASP3, cleaved caspase-3; E10.5, embryonic day 10.5; E11.5, embryonic day 11.5; EP, embryo proper; IF, immunofluorescence; PECAM, platelet endothelial cell adhesion molecule; RIPK1, receptor interacting serine/threonine kinase 1; TNFR1, tumor necrosis factor receptor-1; YS, yolk sac.

As previously reported, caspase-3 cleavage was easily detected in the yolk sacs of *Ripk1*^*D325A/D325A*^ embryos at embryonic day 9.5 (E9.5) and E10.5 (Figs 7A and S6D). Consistently, TUNEL assay also revealed strong signals early in E9.5 *Ripk1*^*D325A/D325A*^ yolk sacs (S6D Fig), suggesting a role for caspase-3-dependent apoptosis. To test whether caspase-3 is the bona fide executioner here, we generated *Casp3*^*−/−*^ mice (S6E Fig). The same as the *Casp3* knockout mice described by the Jackson Laboratory, *Casp3*^*−/−*^ female mice have intrinsic defects in reproductive system and suboptimal mothering instincts, resulting in a low success rate in timed mating. However, despite the difficulties in pregnancy, normal *Ripk1*^*D325A/D325A*^ *Casp3*^*−/−*^ embryos were obtained at E10.5, and vasculature defects started to appear at E11.5 (Fig 7B and 7C), demonstrating that caspase-3 plays a fundamental role in executing E10.5 RIPK1 D325A mutation-caused lethality. In accordance with this genetic evidence, IF staining showed caspase-3 cleavage in E10.5 yolk sacs of *Ripk1*^*D325A/D325A*^, *Ripk1*^*D325A/D325A*^ *Apaf1*^*−/−*^, *Ripk1*^*D325A/D325A*^ *Casp1*^*−/−*^, *Ripk1*^*D325A/D325A*^ *Casp11*^*−/−*^, and *Ripk1*^*D325A/D325A*^ *Asc*^*−/−*^ embryos (Figs 7A and S6F). In addition, caspase-3 cleavage was largely alleviated by the deficiency of *Tnfr1*, RIPK1 kinase activity, *Ripk3*, *Casp8*, caspase-8 catalytic activity, or *Casp1+11* (Fig 7A), indicating that caspase-3 is the downstream executor of the TNFR1-[RIPK1(D325A)-RIPK3-caspase-8]-caspase-1&caspase-11 death signaling at E10.5 in *Ripk1*^*D325A/D325A*^ mice. Because caspase-8 is the activator of caspase-3 in apoptosis in many types of cells, we assessed the possibility of caspase-8 directly activating caspase-3 by evaluating the level of blockade of caspase-3 cleavage in *Ripk1*^*D325A/D325A*^ *Casp8*^*−/−*^ and *Ripk1*^*D325A/D325A*^ *Casp1+11*^*−/−*^ yolk sacs, respectively. By quantification of cells positive for cleaved caspase-3 staining, we found that there are almost no cleaved caspase-3-positive cells in *Ripk1*^*D325A/D325A*^ *Casp8*^*−/−*^ yolk sacs, while there is a dramatic reduction but not complete elimination of cleaved caspase-3-positive cells in *Ripk1*^*D325A/D325A*^ *Casp1+11*^*−/−*^ yolk sacs (Fig 7D). Thus, we propose that activation of caspase-3 in *Ripk1*^*D325A/D325A*^ yolk sacs is mediated primarily by caspase-1+11 and that there is still a small amount of caspase-3 that is cleaved by caspase-8, but this small amount of cleaved caspase-3 is insufficient to trigger apoptosis (Fig 7E). Since *Casp3* deletion only rescued *Ripk1*^*D325A/D325A*^ mice by 1 day and the *Ripk1*^*D325A/D325A*^ *Casp3*^*−/−*^ embryos still died after E11.5, there might be other executioners compensating the loss of caspase-3. Other executioner caspases such as caspase-6 and caspase-7 might be activated later than caspase-3 in *Ripk1*^*D325A/D325A*^ embryos, and it might be that the sum of their involvements determines the fate of the embryos.

## Discussion

Noncleavable variants of RIPK1 are detrimental in humans, and heterozygous mutations cause an early-onset periodic fever syndrome and severe intermittent lymphadenopathy [28,29]. Homozygous D325A mutation in murine RIPK1 leads to embryonic lethality [15,28,31]. Currently, there are no data on whether homozygous D324 mutations exist in humans or, if present, whether this mutation might cause embryonic lethality. Due to the presence of caspase-10 in humans but not in mice, whether the conclusions derived from murine studies are applicable to humans is unknown. It was reported that caspase-10 inhibits CD95L-induced cell death via impeding caspase-8 activation in death-inducing signaling complex (DISC) and meanwhile promotes CD95L-mediated NF-κB activation and gene induction [64]. Based on these findings, we would propose that the presence of caspase-10 in humans may cause the D324 mutation to drive inflammatory gene induction rather than cell death. This speculation is in line with the observations that patients carrying a D324 mutation had periodic fever syndrome and intermittent lymphadenopathy, overproduction of inflammatory cytokines and chemokines, splenomegaly, hepatomegaly, lymphocyte count/percentage increase, and/or monocyte percentage increase in PBMCs and that these patients responded to the IL-6 inhibitor tocilizumab but did not respond to TNF inhibitors [28,29]. In contrast, *Ripk1*^*D325A/+*^ mice were apparently normal in the absence of exogenous pathologic stresses [15,28,31] but were more sensitive to TNF-induced death [15]. The latter may be attributed in part to the lack of caspase-10 [64].

The earlier embryonic lethality of *Ripk1*^*D325A/D325A*^ mice compared with *Casp8*^*−/−*^ mice demonstrates that the effect of RIPK1 D325A mutation in vivo is not simply due to the loss of RIPK1 cleavage (Fig 1). By using genetic and other approaches, we further elucidated the molecular mechanisms underlying RIPK1 D325A-caused E10.5 lethality. In combination with published data [15,28], a previously unanticipated death signaling mechanism is proposed for the lethality of *Ripk1*^*D325A/D325A*^ mice at E10.5 (Fig 7E). TNFR1 engages RIPK1 (D325A), the kinase activity of which is required for the signaling transduction [15,28] (S4C Fig). RIPK1 (D325A) on one hand interacts with RIPK3 and on the other hand binds to caspase-8 probably via FADD. Distinct from its classic role in promoting necroptosis, RIPK3 acts as a scaffold of RIPK1(D325A) [15,28] (S2E and S4D Figs), similar to the case of RIPK3 inhibitor GSK-872-induced RIPK1–RIPK3 interaction [43]. The more RIPK1(D325A) in the complex, the more caspase-8 would be recruited which subsequently auto-processes itself (Figs 2 and 3). By an unknown mechanism, apoptotic caspase-8 elicits activation of caspase-1 and/or caspase-11, 2 known regulators of pyroptosis (Figs 3–6). However, caspase-1 and caspase-11 in this scenario do not lead to pyroptosis but rather ultimately activate the apoptosis executioner caspase-3 (Fig 7). Thus, the death pathway leading to the E10.5 lethality of *Ripk1*^*D325A/D325A*^ mice features a fusion of components of necroptosis, extrinsic apoptosis, or pyroptosis (Fig 7E). Since the absence of *Casp8*, caspase-8 enzymatic activity, or *Casp3*, rather than the loss of *Mlkl* or *Gsdmd*, prevented E10.5 lethality of mouse embryos bearing *Ripk1*^*D325A/D325A*^ mutation (Figs 2A, 2B, 3A, 3B, 7B, 7C, 6F, 6G, S2C, and S2D), the cause of lethality of *Ripk1*^*D325A/D325A*^ embryos at E10.5 is not necroptosis or pyroptosis but apoptosis, which is typically considered nonimmunogenic and would not lead to necroinflammation.

FADD is an adaptor protein bridging caspase-8 and RIPK1 [65–68]. Because of the requirement of caspase-8 in RIPK1(D325A)-initiated cell death, FADD should be the adaptor protein for the interactions between caspase-8 and RIPK1(D325A). This is in agreement with the observation that either *Casp8* or *Fadd* deletion can rescue *Ripk1*^*D325A/D325A*^ *Mlkl*^*−/−*^ mice to adulthood [15] (Fig 2C). However, a study using “*RIPK1*^*D324A/D324A*^*FADD*^*−/−*^ mice” [31] reported that deletion of *FADD* provides little improvement to the development of *RIPK1*^*D324A/D324A*^ embryos. Since the time of death of *FADD*^*−/−*^ mice, *RIPK1*^*D324A/D324A*^ mice, and *RIPK1*^*D324A/D324A*^*FADD*^*−/−*^ mice reported in this study is all at E12.5, whereas the lethality of *Ripk1*^*D325A/D325A*^ mice and *Fadd*^*−/−*^ mice was reported at E10.5 and E12.5, respectively, by others [15,28,69], the role of FADD in bridging RIPK1(D325A) and caspase-8 requires additional studies.

Under certain conditions, ASC is required for caspase-8 and caspase-1 interaction [45,59]. However, loss of *Asc* is unable to prevent defects of *Ripk1*^*D325A/D325A*^ embryos (Fig 6D and 6E), suggesting that caspase-8 activates caspase-1 in an alternative way at E10.5 in *Ripk1*^*D325A/D325A*^ mice. The phenomenon that caspase-1 activation requires caspase-8 but is independent of inflammasome components or the adaptor ASC was also reported in *Yersinia* infection of bone marrow–derived macrophages (BMDMs) [52]. Similarly, activation of caspase-11 by caspase-8 was observed in BMDMs infected with *Citrobacter rodentium* or *Escherichia coli* [50]. Thus, the signal transduction we revealed here from caspase-8 to caspase-1 and/or caspase-11 is not exclusive during embryogenesis and might be a common mechanism occurring in many biological processes.

As intrinsic apoptosis is not required for the death of *Ripk1*^*D325A/D325A*^ embryos (Fig 6A–6C), caspase-9 cleavage in *Ripk1*^*D325A/D325A*^ embryos might be a by-product of activation of TNFR1 signaling and thus is not pathologically relevant. Since previous report showed that caspase-3 can process caspase-9 and other upstream proteins in WT mice following anti-Fas injection [70], the possibility of caspase-9 being one of the substrates of active caspase-3 in *Ripk1*^*D325A/D325A*^ embryos cannot be excluded.

As caspase-1 and caspase-11 are inflammatory caspases usually exerting functions independent of other caspases, it is intriguing to find that caspase-1 and caspase-11 are required for activation of caspase-3 at E10.5 in *Ripk1*^*D325A/D325A*^ mice (Figs 4, 7A, and S6F). The fact that caspase-1 and caspase-11 can be upstream of caspase-3 is supported by observations that caspase-1 initiates apoptosis in the absence of GSDMD [71], that caspase-1, independent of caspase-8, activates caspase-3 and caspase-7, leading to apoptotic features of pyroptosis [72], that caspase-11 is a critical initiator for caspase-3 in a mouse stroke model [73], and that loss of *Casp1* and *Casp11* prevents caspase-3 activation in epidermis in *cpdm* mice [37].

A substantial number of researches employed *Casp1* and *Casp11* double knockout mice to investigate their roles in pathogenesis. The additional genetic tools of *Casp1*^*−/−*^ mice and *Casp11*^*−/−*^ mice help greatly to unravel the overlapping functions and individual contributions of caspase-1 and caspase-11 in vivo. Provided that caspase-1 and caspase-11 can independently process GSDMD for pyroptosis and that caspase-1 can function alone in canonical inflammasomes or downstream of caspase-11 in noncanonical inflammasome activation, it is not surprising to find that caspase-1 and caspase-11 show additive or compensatory effects in some contexts or that one plays a dominant role and is sufficient to exert functions, whereas the other is not required in certain experimental settings [32–35,74–76]. As for the E10.5 lethality of *Ripk1*^*D325A/D325A*^ embryos, caspase-1 and caspase-11 should function in parallel in a mutually compensatory way as only concomitant deletion could block the lethality of *Ripk1*^*D325A/D325A*^ embryos at E10.5 (Fig 4).

MEFs are frequently used as an in vitro system to elucidate the molecular mechanisms of in vivo events, but MEFs are not suitable in analyzing the pathways in which caspase-1 and/or caspase-11 was involved (S5B and S5C Fig). Although D325A mutation promotes cell death in both MEFs (S5B–S5G Fig) and embryos, the type of cell death and the involvement of RIPK1 kinase activity [15,28] and caspase activity [15,28] are not the same. The immunostaining data on yolk sacs where defects occur are consistent with our genetic rescue results and others’ [15,28] as well, providing further evidence that an unexpected signaling mechanism triggers apoptosis in E10.5 *Ripk1*^*D325A/D325A*^ embryos (Fig 7E).

Since strong activation of inflammatory responses was observed in human patients with RIPK1 D324 variants [28,29] and cell death was proposed as the major contributor to cytokine induction in a disease-mimic mouse model [28], one would expect the activation of NF-κB, a major TNFR1-triggered inflammatory signaling regulating inflammatory cytokine production, in human patients. Indeed, increased expression of genes in NF-κB pathway in human patient peripheral blood mononuclear cells (PBMCs) was observed in single-cell RNA sequencing [29]. However, NF-κB signaling was not affected in fibroblasts derived from patient skin biopsies [28] or in certain *Ripk1*^*D325A/D325A*^ mouse cells, including MEFs, BMDMs, and mouse dermal fibroblasts (MDFs) [15,28,31]. We crossed *Ripk1*^*D325A/+*^ mice onto *p50*^*−/−*^ background and found that loss of *p50* brought no effect on RIPK1 D325A mutation-caused lethality (S7A and S7B Fig), suggesting that NF-κB is not required for the E10.5 defect of *Ripk1*^*D325A/D325A*^ mice. To address the role of inflammatory cytokine expression in the E10.5 lethality, we performed yolk sac RNA sequencing. We observed a significant difference in expression profiles between WT and *Ripk1*^*D325A/D325A*^ yolk sacs. The top hit, CXCL10, is a chemokine (S7C and S7D Fig). Quantitative PCR analysis confirmed the significantly higher expression of CXCL10 in *Ripk1*^*D325A/D325A*^ yolk sacs, and this expression is blocked by *Tnfr1* deficiency but not by loss of *Ripk3* (S7E and S7F Fig), indicating that signal downstream of RIPK3 is not involved in CXCL10 production at E10.5. Genetic loss of *Cxcl10* failed to rescue RIPK1 D325A mutation-caused lethality (S7G and S7H Fig), excluding the requirement of CXCL10 in the death of *Ripk1*^*D325A/D325A*^ embryos.

The study described in this report specifically focuses on E10.5 lethality of *Ripk1*^*D325A/D325A*^ mice. It revealed that different from previously identified pronecroptotic function of RIPK1 D325A mutation in cultured cells, this RIPK1 variant gains a new function to arouse an unexpected signal transduction of TNFR1-[RIPK1(D325A)-RIPK3-caspase-8]-caspase-1&11-caspase-3 at E10.5 during embryogenesis, resulting in E10.5 lethality of *Ripk1*^*D325A/D325A*^ mice. Unlike the rescue of *Ripk1*^*D325A/D325A*^ mice by genetic deletion of *Tnfr1* that extended the survival to P10 (S2A and S2B Fig), the rescue by genetic loss of *Ripk3*, *Casp1+Casp11*, or *Casp3* did not extend life beyond E16.5 (Figs 4A, 4B, 7B,7C, S3B, and S3C). Hence, there are other checking processes triggered by TNFR1 that function at embryonic days later than E10.5 to surveil embryonic development. The necroptosis that mediates E11.5 lethality of *Casp8*^*−/−*^ embryos is one such mechanism. We shall emphasize that the role of TNFR1 signaling in embryonic development is far from fully understood and that the signaling pathway shown in Fig 7E is only applicable to RIPK1 D325A mutation-caused mouse embryonic lethality at E10.5.

It is very intriguing that defect of individual components of TNFR1 pathway may result in lethal outcomes, while the TNFR1 signaling pathway as a whole is dispensable for development. For instance, *Casp8*^*−/−*^ or *Ripk1*^*−/−*^ mice cannot survive, but additional *Ripk3* deletion enables the triple knockout mice to be viable and fertile. The E10.5 lethality of *Ripk1*^*D325A/D325A*^ mice is an example that even minor changes can lead to severe outcomes in mice, which can be fully prevented by concomitant loss of *Casp8* and *Mlkl*. On the other hand, these facts also reflect that there is much plasticity in the living organism. It is unclear whether those phenomena are relevant to naturally occurring dysregulations. But given the fact that multiple mechanisms involving TNFR1 signaling exist even within the short time frame of midgestation (E10.5-E13.5), we would believe that TNFR1 functions as a key check mechanism in development, but it is one that is dispensable when there are no major genetic errors.

## Materials and methods

### Ethics statement

All mice were housed in specific pathogen-free condition with 12-hour light/dark cycle and access to food and water ad libitum at Xiamen University Laboratory Animal Center. Animal husbandry and all mouse experiments were reviewed and approved by Laboratory Animal Management and Ethics Committee of Xiamen University (approval number XMULACC20180126) and were in strict accordance with good animal practice as defined by Xiamen University Laboratory Animal Center. Before the embryos were isolated, the pregnant females were euthanized via CO_2_ exposure for at least 5 minutes until no breathing was observed. Death was ensured by performing a toe pinch. Cervical dislocation was performed as a secondary method of euthanasia.

### Mice

*Ripk1*^*D325A/+*^ mice, *Ripk1*^*D325A;△/+*^ mice, *Casp8*^*+/−*^ mice, *Casp3*^*−/−*^ mice, *Casp1*^*−/−*^ mice, *Casp11*^*−/−*^ mice, *Casp1+11*^*−/−*^ mice, *Asc*^*−/−*^ mice, *Apaf1*^*−/−*^ mice, and *Cxcl10*^*−/−*^ mice were generated by Xiamen University Laboratory Animal Center as previously described [77]. gRNA targeting sequence was 5′-GTGTACCCTTACCTCCGAGC-3′ for *Ripk1* D325A mutation, 5′-CCCGAAGCCTCCGCTGTCT-3′ for *Ripk1* △ mutation (F28G29 deletion, exactly the same mutation form as the original publication) [41], 5′-TTCCTAGACTGCAACCGAG-3′ for *Casp8* knockout, 5′- AGTGGACTCTGGGATCTATC -3′ for *Casp3* knockout, 5′-GGGACAATAAATGGATTGTTGG-3′ for *Casp1* knockout in *Casp1*^*−/−*^ mice and *Casp1+11*^*−/−*^ mice, 5′-GCCAATGGCCGTACACGAAAGG-3′ and 5′-GACTTAGGCTACGATGTGG-3′for *Casp11* knockout in *Casp11*^*−/−*^ mice and *Casp1+11*^*−/−*^ mice, 5′-TATGGGCGCATCCCACGCG-3′ for *Asc* knockout, 5′-TGGCGTCTTGTCAGTGATAG −3′ for *Apaf1* knockout, and 5′-GAGTCCCACTCAGACCCAGC-3′ and 5′-AGCGGACCGTCCTTGCGAGA-3′ for *Cxcl10* knockout. *Casp8*^*C362S/+*^ mice, *Ripk3*^*−/−*^ mice, *Mlkl*^*−/−*^ mice, and *Gsdmd*^*−/−*^ mice were generated as described previously [48,78,79]. *p50*^*−/−*^ mice [80] and *Tnfr1*^*−/−*^ mice [2] were from the Jackson Laboratory (JAX stock #002849 and #002818). All knockout/knockin alleles have been crossed onto the C57BL/6J background, and mice with H19 and DMR mutations were excluded by using PCR as previously reported [77]. Additional information is provided upon request.

### Cell culture

All MEFs were harvested from E10.5 embryos. All cells were cultured in Dulbecco’s Modified Eagle Medium (Life Technologies, NY, USA) supplemented with 10% fetal bovine serum (Life Technologies, NSW, Australia), 1% MEM nonessential amino acids solution (Life Technologies, NY, USA), and 100 units/mL penicillin/streptomycin, at 37°C in a humidified incubator containing 5% CO2. All cell lines were well established and frequently checked by monitoring morphology and functionalities. All the cell lines were authenticated by STR analysis and were routinely tested to be mycoplasma free.

### Timed mating analysis and imaging

For timed mating experiments, 3- to 4-month-old stud males were housed individually for 1 to 2 weeks prior to mating. Females that are 8 to 15 weeks old were group housed for 10 to 14 days prior to mating and were exposed to soiled bedding from a male’s cage 2 days before mating. One to 2 female(s) in estrus were added into each stud male’s cage at 9:00 PM. Females were examined for vaginal plugs and were separated from male mice at 9:00 AM the next morning. The morning when a vaginal plug was found was set as embryonic day 0.5 (E0.5). At 9:00 AM in the morning of E10.5 or other embryonic stages as indicated, pregnant females were killed. Embryos were isolated, and images of yolk sacs and embryo proper were captured on Leica M165FC microscope (Leica Microsystems GmbH, Germany).

### Antibody

Antibodies for IF staining: CD31 (BD Biosciences, 550274); cleaved caspase-3 (Cell Signaling Technology, 9661S); cleaved caspase-8 (Cell Signaling Technology, 9429S); cleaved caspase-1 (Affbiotech, AF4005); cleaved caspase-9 (Cell Signaling Technology, 9509S); cleaved GSDMD (Cell Signaling Technology, 50928S); ASC (Cell Signaling Technology, 67824S); phospho-RIPK3 (2D7) (T231, S232) (Abcam, ab205421); phospho-MLKL (S345) (Abcam, ab196436); Alexa Fluor 488 goat anti-mouse antibody (Invitrogen, A11029); Alexa Fluor 594 goat anti-rat antibody (Invitrogen, A11007); and Alexa Fluor 488 goat anti-rabbit antibody (Invitrogen, A11034). Antibodies for western blot: Apaf-1 (Cell Signaling Technology, 8723S); caspase-3 (Cell Signaling Technology, 9662); caspase-1 (clone 4B4, a kind gift from Dr. Vishva M. Dixit, Genetech, United States of America); caspase-11 (Cell Signaling Technology, 14340S); caspase-8 (Cell Signaling Technology, 4790S); RIPK1 (Cell Signaling Technology, 3493S); TNFR1 (Proteintech, 21514-1-AP); TRADD (Abcam, ab110644-100 uL); A20 (Cell Signaling Technology, 5630S); SHARPIN (Proteintech, 14626-1-AP); RIPK3 (Cell Signaling Technology, 13526); FADD (Proteintech, 14906-1-AP); anti-GSDMA polyclonal antibodies (Immunogen: GST-GSDMA (248-347aa)) and anti-GSDMC polyclonal antibodies (Immunogen: GST-GSDMC2 (367-467aa)) were raised in rabbits; GSDMD (Abcam, ab209845); and GSDME (Abcam, ab215191). Antibodies for immunoprecipitation: RIPK1 (BD Biosciences, 610459).

### Immunofluorescence staining and confocal microscopy

Yolk sacs were harvested, mounted on adhesion microscope slides (ZSGB-Bio), and fixed for 4 hours at 4°C using 4% paraformaldehyde in PBS. Cells were permeabilized for 45 minutes in 0.25% Triton X-100 in PBS, blocked for 1 hour in PBS containing 2% goat serum, and then incubated overnight at 4°C with rabbit anti-cleaved caspase-3, rabbit anti-cleaved caspase-1, rabbit anti-cleaved caspase-8, mouse anti-phospho-RIPK3 (2D7), or rabbit anti-phospho-MLKL (S345) along with PECAM (CD31) diluted in blocking buffer. Yolk sacs were washed 3 times with PBS and then incubated for 1 hour at room temperature with Alexa Fluor 488 goat anti-mouse antibody (Invitrogen, A11029), Alexa Fluor 594 goat anti-rat antibody (Invitrogen, A11007), or Alexa Fluor 488 goat anti-rabbit antibody (Invitrogen, A11034). TUNEL assay was performed with commercially available kit (Promega, G3250). DAPI was used as a nuclear (DNA) counter stain. Images were acquired on a Zeiss LSM 780 laser scanning confocal microscope (Carl Zeiss Microscopy GmbH, Germany).

### Cell death assay

Cell death was analyzed using CellTiter-Glo luminescent cell viability assay kit (Promega, WI, USA). The luminescent cell viability assays were performed according to the manufacturer’s instructions. In brief, 2.0 × 10^5^ cells were seeded in 96-well plates with white wall. After treatment, an equal volume of CellTiter-Glo reagent was added to the cell culture medium, which had been equilibrated to room temperature for 30 minutes. Cells were shaken for 5 minutes and incubated at room temperature for 15 minutes. Luminescent recording was performed with POLAR star Omega (BMG Labtech, Durham, North Carolina, USA).

### Immunoprecipitation

Cell pellets were collected in ice-cold PBS and resuspended in lysis buffer (12.5 mM HEPES (pH7.5), 30 mM NaCl, 90 mM NaSCN, 1% NP40, and protease inhibitor cocktail). The resuspended cell pellets were sonicated and centrifuged at 20,000*g* for 30 minutes at 4°C. The supernatants were collected for immunoblotting or immunoprecipitation. Commercially available anti-Flag beads were used in the immunoprecipitation. For immunoprecipitation of endogenous proteins, antibody coupled beads were made as follows. Antibodies were crosslinked to A/G agarose beads for 8 hours at 4°C. The cell lysates were then incubated with the antibody-coupled beads overnight at 4°C. The beads were washed with lysis buffer, and the immunoprecipitates of anti-Flag beads were eluted off the beads with 3X FLAG peptide. To minimize the influence of IgG in immunoblotting, the Rabbit TrueBlot was used.

### Western blot

Tissues were collected in RIPA buffer (20 mM Tris-HCl (pH7.5), 150 mM NaCl, 1 mM Na_2_EDTA, 1 mM EGTA, 1% NP40, 1% sodium deoxycholate, 2.5 mM sodium pyrophosphate, 1 mM β-glycerophosphate, 1 mM Na_3_VO_4_, 1 μg/mL leupeptin, and protease inhibitor cocktail) and were mechanically homogenized with a bead mill homogenizer (Scientz-IID, SCIENTZ, Ningbo, China). Insoluble pellet was removed by centrifugation at 20,000*g* before addition of SDS sample buffer. The supernatants were collected for immunoblotting.

## Supporting information

S1 Fig The embryonic lethality caused by D325A mutation in *Ripk1* occurs earlier than that triggered by *Casp8* knockout in mice. **Related to Fig 1.** (A) Western blot analysis for caspase-8 expression in E12.5 embryos from intercrosses of *Casp8*^*+/−*^ mice and for RIPK1 expression in E10.5 embryos from intercrosses of *Ripk1*^*D325A/+*^ mice. (B) Genetic analysis of offspring from timed mating of *Ripk1*^*D325A/+*^ and *Ripk1*^*+/−*^ parents. ♦: paler and likely defective embryos, which are shown in (C). (C) Representative E10.5 and E17.5 embryos in (B). Scale bars, 1 mm. (D) Western blot analysis for RIPK1 expression in E10.5 embryos. Genotypes are as indicated. Uncropped immunoblot for panels A and D can be found in S1 Raw Images. E10.5, embryonic day 10.5; E12.5, embryonic day 12.5; E17.5, embryonic day 17.5; EP, embryo proper; GAPDH, glyceraldehyde 3-phosphate dehydrogenase; RIPK1, receptor interacting serine/threonine kinase 1; YS, yolk sac.
(TIF)

S2 Fig Defects of *Ripk1*^*D325A/D325A*^ mice at E10.5 are mediated by TNFR1 but not MLKL. **Related to Fig 2.** (A) Genetic analysis of offspring from timed mating of *Ripk1*^*D325A/+*^ *Tnfr1*^*−/−*^ parents. ※: defective and likely dead E19.5 embryos. ♣: the P10 homozygous runts. (B) Representative E19.5 embryos and P10 pups described in (A). (C) Genetic analysis of offspring from intercrosses of *Ripk1*^*D325A/+*^ *Mlkl*^*−/−*^ mice. #: defective vascularization in YS and normal EP. ##: no vessels in YS and abdominal hemorrhage in the EP. ###: dead embryos, which were about to be resorbed. (D) Representative E10.5, E11.5, and E12.5 embryos in (C). Scale bars, 1 mm. (E) IF staining of E9.5 and E10.5 YS of indicated genotypes with anti-PECAM (red) and anti-p-MLKL (green) antibodies. Scale bars, 50 μm. Images are representative of 3 embryos per genotype. E9.5, embryonic day 9.5; E10.5, embryonic day 10.5; E11.5, embryonic day 11.5; E12.5, embryonic day 12.5; E19.5, embryonic day 19.5; EP, embryo proper; IF, immunofluorescence; MLKL, mixed lineage kinase domain-like; PECAM, platelet endothelial cell adhesion molecule; p-MLKL, phosphorylated MLKL; TNFR1, tumor necrosis factor receptor-1; YS, yolk sac.
(TIF)

S3 Fig Defects of *Ripk1*^*D325A/D325A*^ mice at E10.5 are mediated by RIPK3 and caspase-8. **Related to Fig 2.** (A) Representative spleens and lymph nodes isolated from WT mice, *Casp8*^*−/−*^ *Mlkl*^*−/−*^ mice, and *Ripk1*^*D325A/D325A*^ *Casp8*^*−/−*^ *Mlkl*^*−/−*^ mice at P48 when the mice began to show *lpr* phenotypes and at 3 months old when symptoms were severe and the mice shall be killed. (B) Genetic analysis of offspring from intercrosses of *Ripk1*^*D325A/+*^ *Ripk3*^*−/−*^ parents. ✿: YS vascularization defect and normal EP; ✿✿: YS vascularization defect, smaller and paler EP. ✿✿✿: dead embryos being resorbed; ✿✿✿✿: dead embryos. (C) Representative E13.5, E15.5, E16.5, E17.5, and E19.5 embryos summarized in (B). Scale bars, 1 mm. E10.5, embryonic day 10.5; E13.5, embryonic day 13.5; E15.5, embryonic day 15; E16.5, embryonic day 16.5; E17.5, embryonic day 17.5; E19.5, embryonic day 19.5; EP, embryo proper; P48, postnatal day 48; RIPK3, receptor interacting serine/threonine kinase 3; WT, wild-type; YS, yolk sac.
(TIF)

S4 Fig Enzymatic activity of caspase-8 and RIPK1 but not RIPK3 is required for E10.5 lethality of *Ripk1*^*D325A/D325A*^ mice. **Related to Fig 3.** (A) Genetic analysis of offspring from intercrosses of *Casp8*^*C362S/+*^ mice. *: defective YS vessels in E11.5 embryos. **: severe YS vascularization defect and dead EP in E12.5 embryos. (B) Representative E10.5, E11.5, and E12.5 embryos obtained in (A). Scale bars, 1 mm. (C) Genetic analysis of progeny from intercrossing *Ripk1*^*D325A; Δ/+*^ parents. (D) IF staining of E10.5 YS of indicated genotypes with anti-PECAM (red) and anti-p-RIPK3 (green) antibodies. Scale bars, 50 μm. Images are representative of 3 embryos per genotype. E10.5, embryonic day 10.5; E11.5, embryonic day 11.5; E12.5, embryonic day 12.5; EP, embryo proper; IF, immunofluorescence; PECAM, platelet endothelial cell adhesion molecule; p-RIPK3, phosphorylated RIPK3; RIPK1, receptor interacting serine/threonine kinase 1; YS, yolk sac.
(TIF)

S5 Fig Concurrent deficiency of *Casp1* and *Casp11* is required for prevention of E10.5 lethality of *Ripk1*^*D325A/D325A*^ mice, and caspase-8 is upstream of caspase-1 in the death pathway. (A) Western blot analysis of caspase-1 and/or caspase-11 expression in tissues from the respective knockout mice. Related to Fig 4. (B) MEFs of indicated genotypes were treated with TNF (10 ng/mL) for different periods of time, and cell death was measured. Data are represented as mean ± SD of triplicates. (C) MEFs of indicated genotypes were treated with TNF (10 ng/mL) for different periods of time. Expression levels of caspase-8, caspase-3, RIPK1, RIPK3, caspase-1, and caspase-11 were analyzed by western blot. (D) *Ripk1*^*+/+*^ and *Ripk1*^*D325A/D325A*^ MEFs were treated with 3*FLAG-tagged TNF (3*FLAG-TNF, 200 ng/mL) for different periods of time. Cell lysates were subjected to immunoprecipitation with mouse anti-FLAG M2 beads and then western blotting with anti-RIPK1, anti-TNFR1, anti-TRADD, anti-A20, and anti-SHARPIN antibodies as indicated. (E–G) *Ripk1*^*+/+*^ and *Ripk1*^*D325A/D325A*^ MEFs were treated with TNF (10 ng/mL), TS (10 μM), or TSZ (20 μM) for indicated periods of time. Cell lysates were subjected to IP with anti-RIPK1 antibody and then western blotting with anti-RIPK1, anti-caspase-8, anti-RIPK3, and anti-FADD antibodies. *: nonspecific band. Panels B–G are related to Fig 5. Uncropped immunoblot for panels A and C–G can be found in S1 Raw Images. Underlying data are available in S1 Data. E10.5, embryonic day 10.5; FADD, FAS-associated death domain protein; GAPDH, glyceraldehyde 3-phosphate dehydrogenase; IP, immunoprecipitation; MEF, mouse embryonic fibroblast; RIPK1, receptor interacting serine/threonine kinase 1; SHARPIN, SHANK-associated RH domain interacting protein; TNF, tumor necrosis factor; TNFR1, tumor necrosis factor receptor-1; TRADD, TNFR1-associated death domain protein; TS, TNF + SMAC mimetic; TSZ, TNF + SMAC mimetic + zVAD; WT, wild-type.
(TIF)

S6 Fig Caspase-3 participates in the E10.5 lethality of RIPK1 D325A mutant mice while neither intrinsic apoptosis nor pyroptosis is required. (A) Western blot analysis for Apaf-1 expression in embryos of indicated genotypes. (B) IF staining of E10.5 YS of indicated genotypes with anti-PECAM (red) and anti-ASC (green) or anti-Cl.GSDMD (green) antibodies. Scale bars, 50 μm. Images are representative of 3 embryos per genotype. (C) Western blot analysis for expression of gasdermins in E10.5 WT mouse embryos. Antibodies against the conserved region of 3 GSDMAs and conserved region of 4 GSDMCs, GSDMD, and GSDME were used. Anti-RIPK1, anti-caspase-8, and anti-caspase-3 antibodies were included as controls. *: nonspecific band. Panels A–C are related to Fig 6. (D) TUNEL assay of E9.5 YS and IF staining of E9.5 YS with anti-PECAM (red) and anti-Cl.CASP3 (green) antibodies. DAPI, a DNA stain. Scale bars, 50 μm. Images are representative of 3 embryos per genotype. (E) Western blot analysis for caspase-3 expression in different tissues from mice of indicated genotypes. (F) IF staining of E10.5 YS of indicated genotypes with anti-PECAM (red) and anti-Cl.CASP3 (green) antibodies. Scale bars, 50 μm. Images are representative of 3 embryos per genotype. Panels D–F are related to Fig 7. Uncropped immunoblot for panels A, C, and E can be found in S1 Raw Images. Apaf-1, apoptotic protease activating factor 1; ASC, apoptosis-associated speck-like protein containing a CARD; BMDM, bone marrow–derived macrophage; Cl.CASP3, cleaved caspase-3; Cl.GSDMD, cleaved GSDMD; E10.5, embryonic day 10.5; EP, embryo proper; GAPDH, glyceraldehyde 3-phosphate dehydrogenase; IF, immunofluorescence; PECAM, platelet endothelial cell adhesion molecule; RIPK1, receptor interacting serine/threonine kinase 1; TUNEL, terminal deoxynucleotidyltransferase-mediated dUTP-biotin nick end labeling; WT, wild-type; YS, yolk sac.
(TIF)

S7 Fig NF-κB-mediated inflammatory response is not required for E10.5 lethality of *Ripk1*^*D325A/D325A*^ mice. (A) Genetic analysis of offspring from intercrosses of *Ripk1*^*D325A/+*^ *p50*^*−/−*^ parents. (B) Representative E10.5 embryos collected in (A). Scale bars, 1 mm. (C) RNA sequencing of YS RNA from WT and *Ripk1*^*D325A/D325A*^ mice, both with biological triplicates. Heat map shows differentially expressed genes. (D) A list of top 10 high expression genes along with their parameters and values analyzed in (C). (E and F) Quantitative PCR analysis of CXCL10 expression in E10.5 YS. Data from 3 independent experiments are analyzed, 3 embryos per genotype per experiment. Data are represented as mean ± SEM. ns, no significance; ****: *p* < 0.0001; **: *p* < 0.01; *: *p* < 0.05. (G) Genetic analysis of progeny from intercrossing *Ripk1*^*D325A/+*^ *Cxcl10*^*−/−*^ mice. (H) Representative images of E11.5 embryos obtained in (G). Scale bars, 1 mm. Underlying data are available in S1 Data. E10.5, embryonic day 10.5; E11.5, embryonic day 11.5; EP, embryo proper; HM *Ripk1^D325A/D325A^* homozygotes; NF-κB, nuclear factor-kappa B; WT, wild-type; YS, yolk sac.
(TIF)

S1 Data Excel file containing numerical values that underlie the summary data displayed in Figs 7D, S5B, S7C, S7E and S7F. (XLSX)

S1 Raw Images Uncropped immunoblot images for S1A and S1D Fig, S5A, S5C-S5G Fig, S6A, S6C, and S6E Fig. (PDF)

## Abbreviations

Apaf-1: apoptotic protease activating factor 1
ASC: apoptosis-associated speck-like protein containing a CARD
BMDM: bone marrow–derived macrophage
cFLIP: CASP8 and FADD-like apoptosis regulator
cIAP1 and 2: cellular inhibitor of apoptosis protein 1 and 2
DISC: death-inducing signaling complex
E9.5: embryonic day 9.5
E10.5: embryonic day 10.5
E11.5: embryonic day 11.5
E12.5: embryonic day 12.5
E13.5: embryonic day 13.5
E16.5: embryonic day 16.5
E17.5: embryonic day 17.5
FADD: FAS-associated death domain protein
HOIL-1: RanBP-type and C3HC4-type zinc finger-containing protein 1
HOIP: ring finger protein 31
IF: immunofluorescence
IKKβ: inhibitor of nuclear factor kappa-B kinase subunit beta
LUBAC: linear ubiquitin chain assembly complex
MAPK: mitogen-activated protein kinase
MDF: mouse dermal fibroblast
MEF: mouse embryonic fibroblast
MLKL: mixed lineage kinase domain-like
NF-κB: nuclear factor-kappa B
P1: postnatal day 1
P10: postnatal day 10
PBMC: peripheral blood mononuclear cell
RHIM: RIP homotypic interaction motif
RIPK1: receptor interacting serine/threonine kinase 1
SHARPIN: SHANK-associated RH domain interacting protein
TBK1: TANK-binding kinase 1
TNF: tumor necrosis factor
TNFR1: tumor necrosis factor receptor-1
TRADD: TNFR1-associated death domain protein
TS: TNF + SMAC mimetic
TSZ: TNF + SMAC mimetic + zVAD
WT: wild-type
