# Supplementary figures and images for: A unique death pathway keeps RIPK1 D325A mutant mice in check at embryonic day 10.5
Source: PLoS Biol. 2021 Aug 26;19(8):e3001304. doi: 10.1371/journal.pbio.3001304 (PMC8389420; doi:10.1371/journal.pbio.3001304)

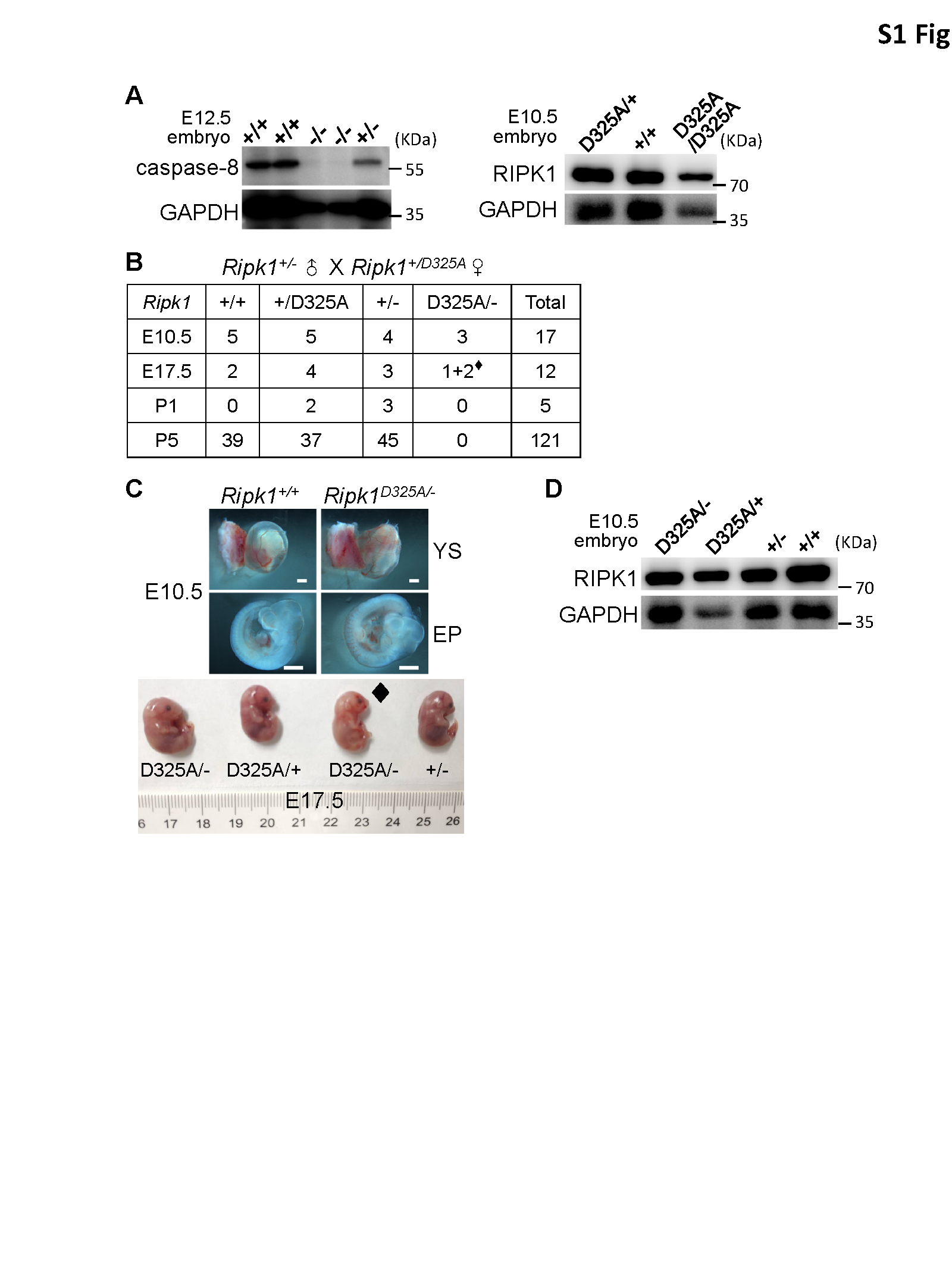

Supplement: S1 Fig — Related to Fig 1. (A) Western blot analysis for caspase-8 expression in E12.5 embryos from intercrosses of Casp8+/− mice and for RIPK1 expression in E10.5 embryos from intercrosses of Ripk1D325A/+ mice. (B) Genetic analysis of offspring from timed mating of Ripk1D325A/+ and Ripk1+/− parents. ♦: paler and likely defective embryos, which are shown in (C). (C) Representative E10.5 and E17.5 embryos in (B). Scale bars, 1 mm. (D) Western blot analysis for RIPK1 expression in E10.5 embryos. Genotypes are as indicated. Uncropped immunoblot for panels A and D can be found in S1 Raw Images. E10.5, embryonic day 10.5; E12.5, embryonic day 12.5; E17.5, embryonic day 17.5; EP, embryo proper; GAPDH, glyceraldehyde 3-phosphate dehydrogenase; RIPK1, receptor interacting serine/threonine kinase 1; YS, yolk sac. (TIF) [file pbio.3001304.s001.tif]

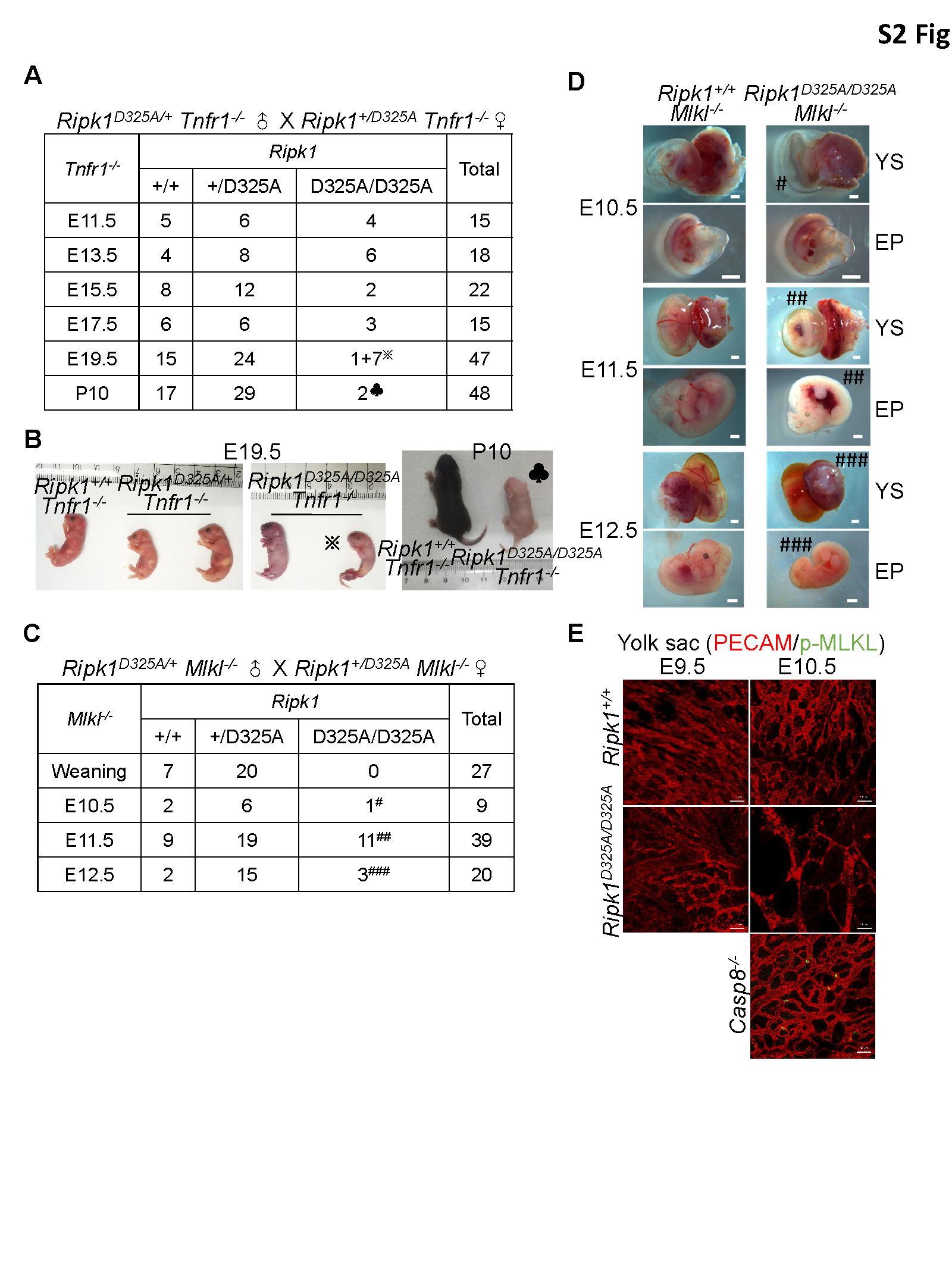

Supplement: S2 Fig — Related to Fig 2. (A) Genetic analysis of offspring from timed mating of Ripk1D325A/+ Tnfr1−/− parents. ※: defective and likely dead E19.5 embryos. ♣: the P10 homozygous runts. (B) Representative E19.5 embryos and P10 pups described in (A). (C) Genetic analysis of offspring from intercrosses of Ripk1D325A/+ Mlkl−/− mice. #: defective vascularization in YS and normal EP. ##: no vessels in YS and abdominal hemorrhage in the EP. ###: dead embryos, which were about to be resorbed. (D) Representative E10.5, E11.5, and E12.5 embryos in (C). Scale bars, 1 mm. (E) IF staining of E9.5 and E10.5 YS of indicated genotypes with anti-PECAM (red) and anti-p-MLKL (green) antibodies. Scale bars, 50 μm. Images are representative of 3 embryos per genotype. E9.5, embryonic day 9.5; E10.5, embryonic day 10.5; E11.5, embryonic day 11.5; E12.5, embryonic day 12.5; E19.5, embryonic day 19.5; EP, embryo proper; IF, immunofluorescence; MLKL, mixed lineage kinase domain-like; PECAM, platelet endothelial cell adhesion molecule; p-MLKL, phosphorylated MLKL; TNFR1, tumor necrosis factor receptor-1; YS, yolk sac. (TIF) [file pbio.3001304.s002.tif]

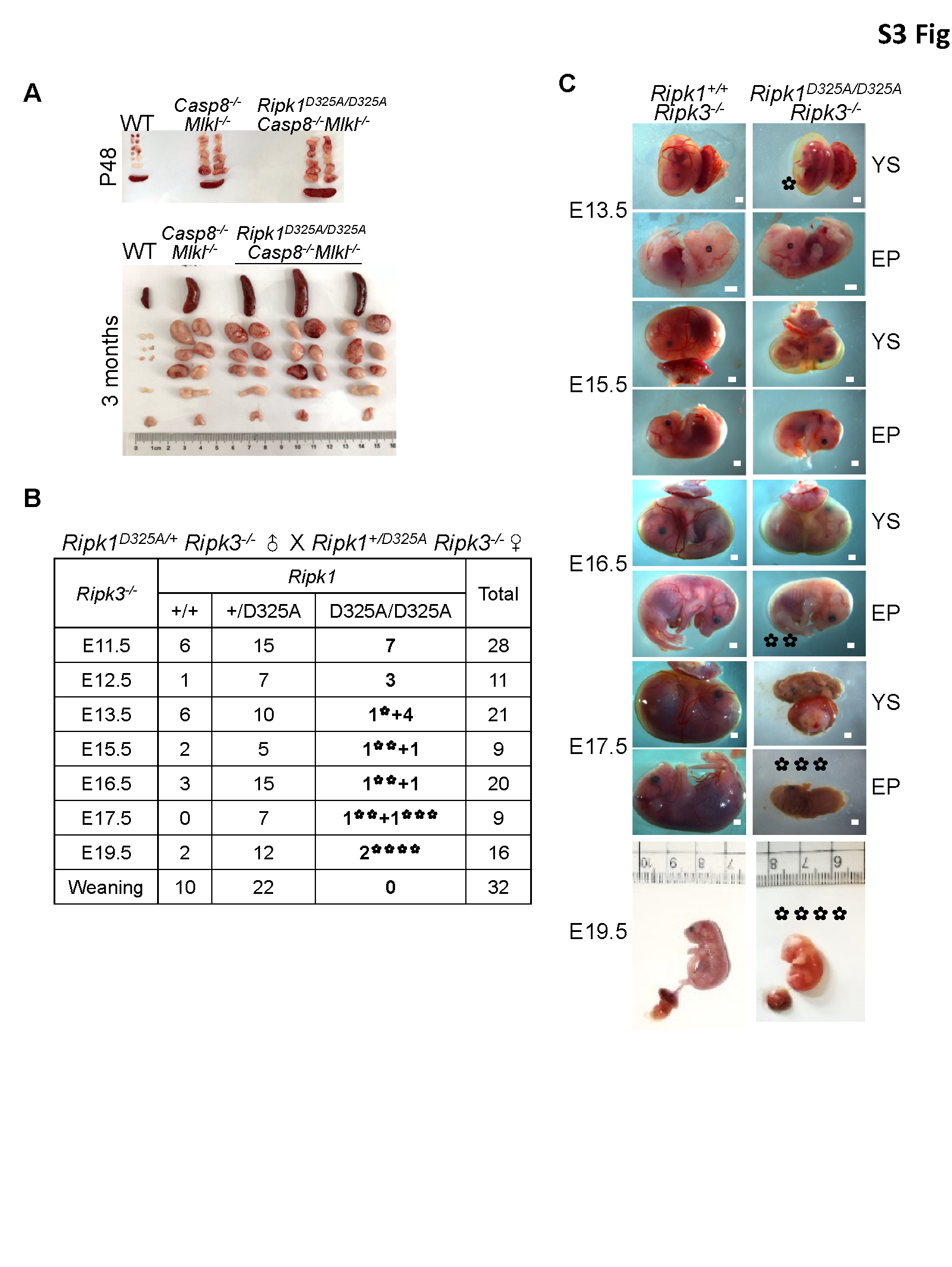

Supplement: S3 Fig — Related to Fig 2. (A) Representative spleens and lymph nodes isolated from WT mice, Casp8−/− Mlkl−/− mice, and Ripk1D325A/D325A Casp8−/− Mlkl−/− mice at P48 when the mice began to show lpr phenotypes and at 3 months old when symptoms were severe and the mice shall be killed. (B) Genetic analysis of offspring from intercrosses of Ripk1D325A/+ Ripk3−/− parents. ✿: YS vascularization defect and normal EP; ✿✿: YS vascularization defect, smaller and paler EP. ✿✿✿: dead embryos being resorbed; ✿✿✿✿: dead embryos. (C) Representative E13.5, E15.5, E16.5, E17.5, and E19.5 embryos summarized in (B). Scale bars, 1 mm. E10.5, embryonic day 10.5; E13.5, embryonic day 13.5; E15.5, embryonic day 15; E16.5, embryonic day 16.5; E17.5, embryonic day 17.5; E19.5, embryonic day 19.5; EP, embryo proper; P48, postnatal day 48; RIPK3, receptor interacting serine/threonine kinase 3; WT, wild-type; YS, yolk sac. (TIF) [file pbio.3001304.s003.tif]

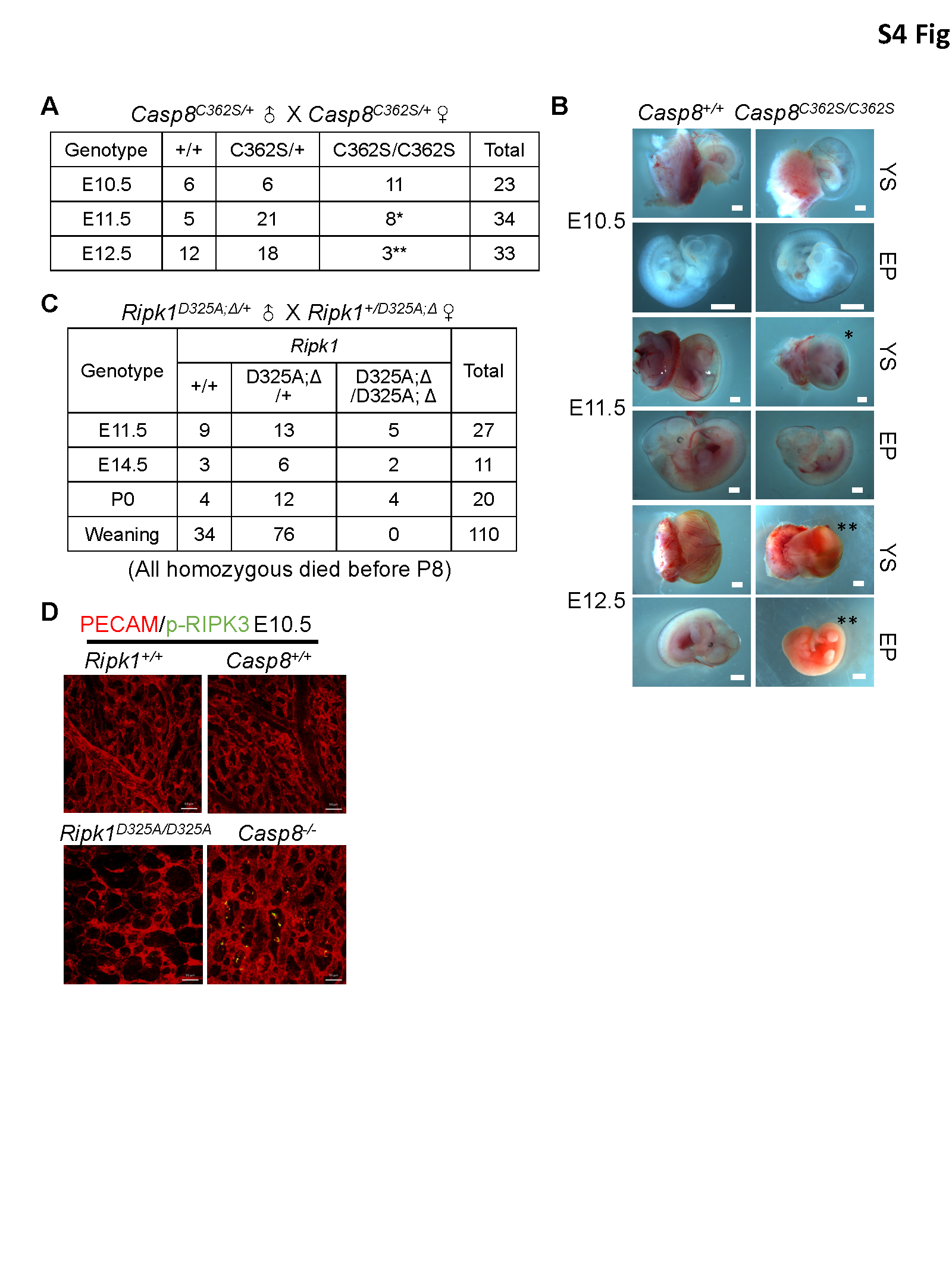

Supplement: S4 Fig — Related to Fig 3. (A) Genetic analysis of offspring from intercrosses of Casp8C362S/+ mice. *: defective YS vessels in E11.5 embryos. **: severe YS vascularization defect and dead EP in E12.5 embryos. (B) Representative E10.5, E11.5, and E12.5 embryos obtained in (A). Scale bars, 1 mm. (C) Genetic analysis of progeny from intercrossing Ripk1D325A; Δ/+ parents. (D) IF staining of E10.5 YS of indicated genotypes with anti-PECAM (red) and anti-p-RIPK3 (green) antibodies. Scale bars, 50 μm. Images are representative of 3 embryos per genotype. E10.5, embryonic day 10.5; E11.5, embryonic day 11.5; E12.5, embryonic day 12.5; EP, embryo proper; IF, immunofluorescence; PECAM, platelet endothelial cell adhesion molecule; p-RIPK3, phosphorylated RIPK3; RIPK1, receptor interacting serine/threonine kinase 1; YS, yolk sac. (TIF) [file pbio.3001304.s004.tif]

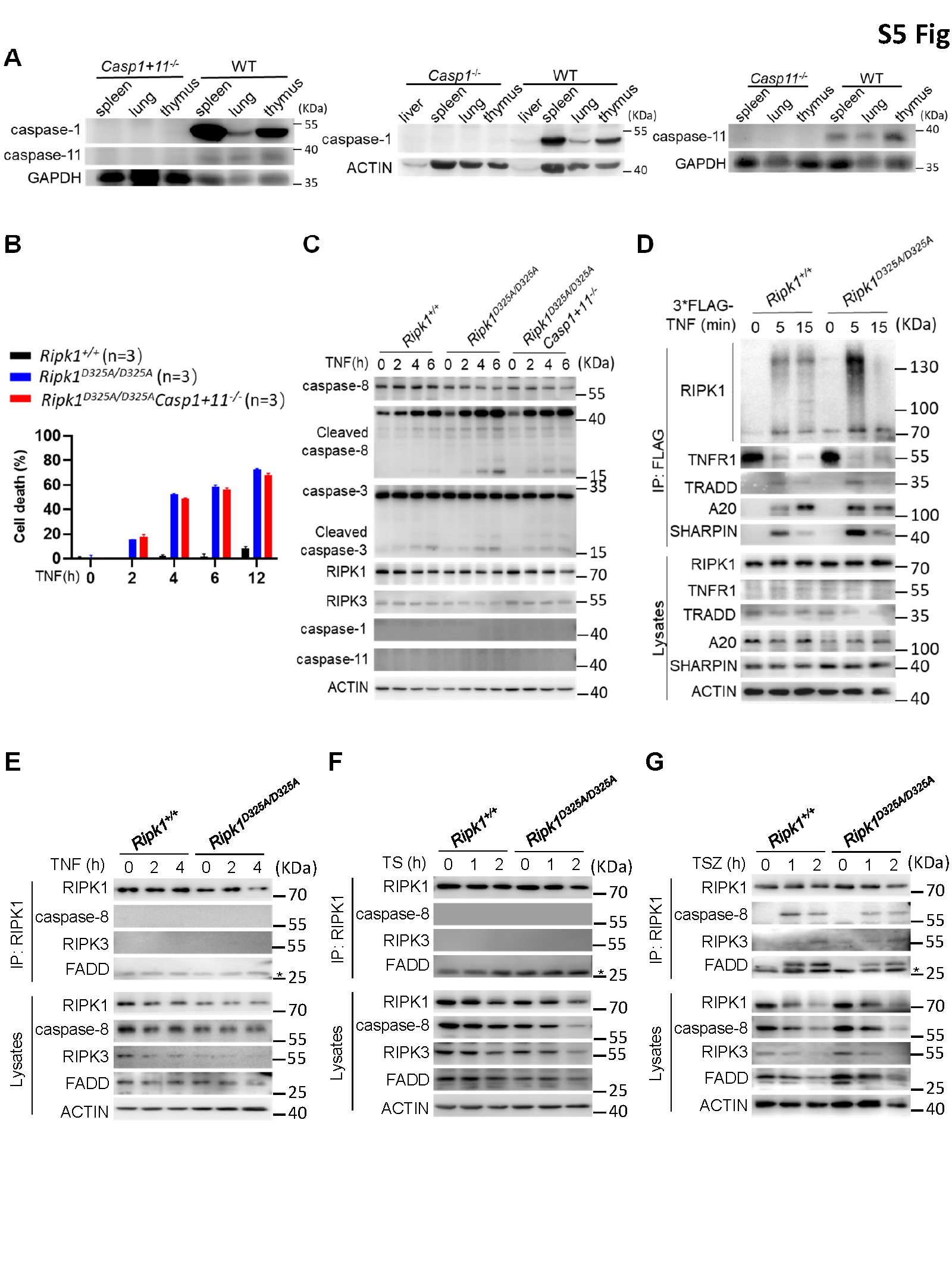

Supplement: S5 Fig — (A) Western blot analysis of caspase-1 and/or caspase-11 expression in tissues from the respective knockout mice. Related to Fig 4. (B) MEFs of indicated genotypes were treated with TNF (10 ng/mL) for different periods of time, and cell death was measured. Data are represented as mean ± SD of triplicates. (C) MEFs of indicated genotypes were treated with TNF (10 ng/mL) for different periods of time. Expression levels of caspase-8, caspase-3, RIPK1, RIPK3, caspase-1, and caspase-11 were analyzed by western blot. (D) Ripk1+/+ and Ripk1D325A/D325A MEFs were treated with 3*FLAG-tagged TNF (3*FLAG-TNF, 200 ng/mL) for different periods of time. Cell lysates were subjected to immunoprecipitation with mouse anti-FLAG M2 beads and then western blotting with anti-RIPK1, anti-TNFR1, anti-TRADD, anti-A20, and anti-SHARPIN antibodies as indicated. (E–G) Ripk1+/+ and Ripk1D325A/D325A MEFs were treated with TNF (10 ng/mL), TS (10 μM), or TSZ (20 μM) for indicated periods of time. Cell lysates were subjected to IP with anti-RIPK1 antibody and then western blotting with anti-RIPK1, anti-caspase-8, anti-RIPK3, and anti-FADD antibodies. *: nonspecific band. Panels B–G are related to Fig 5. Uncropped immunoblot for panels A and C–G can be found in S1 Raw Images. Underlying data are available in S1 Data. E10.5, embryonic day 10.5; FADD, FAS-associated death domain protein; GAPDH, glyceraldehyde 3-phosphate dehydrogenase; IP, immunoprecipitation; MEF, mouse embryonic fibroblast; RIPK1, receptor interacting serine/threonine kinase 1; SHARPIN, SHANK-associated RH domain interacting protein; TNF, tumor necrosis factor; TNFR1, tumor necrosis factor receptor-1; TRADD, TNFR1-associated death domain protein; TS, TNF + SMAC mimetic; TSZ, TNF + SMAC mimetic + zVAD; WT, wild-type. (TIF) [file pbio.3001304.s005.tif]

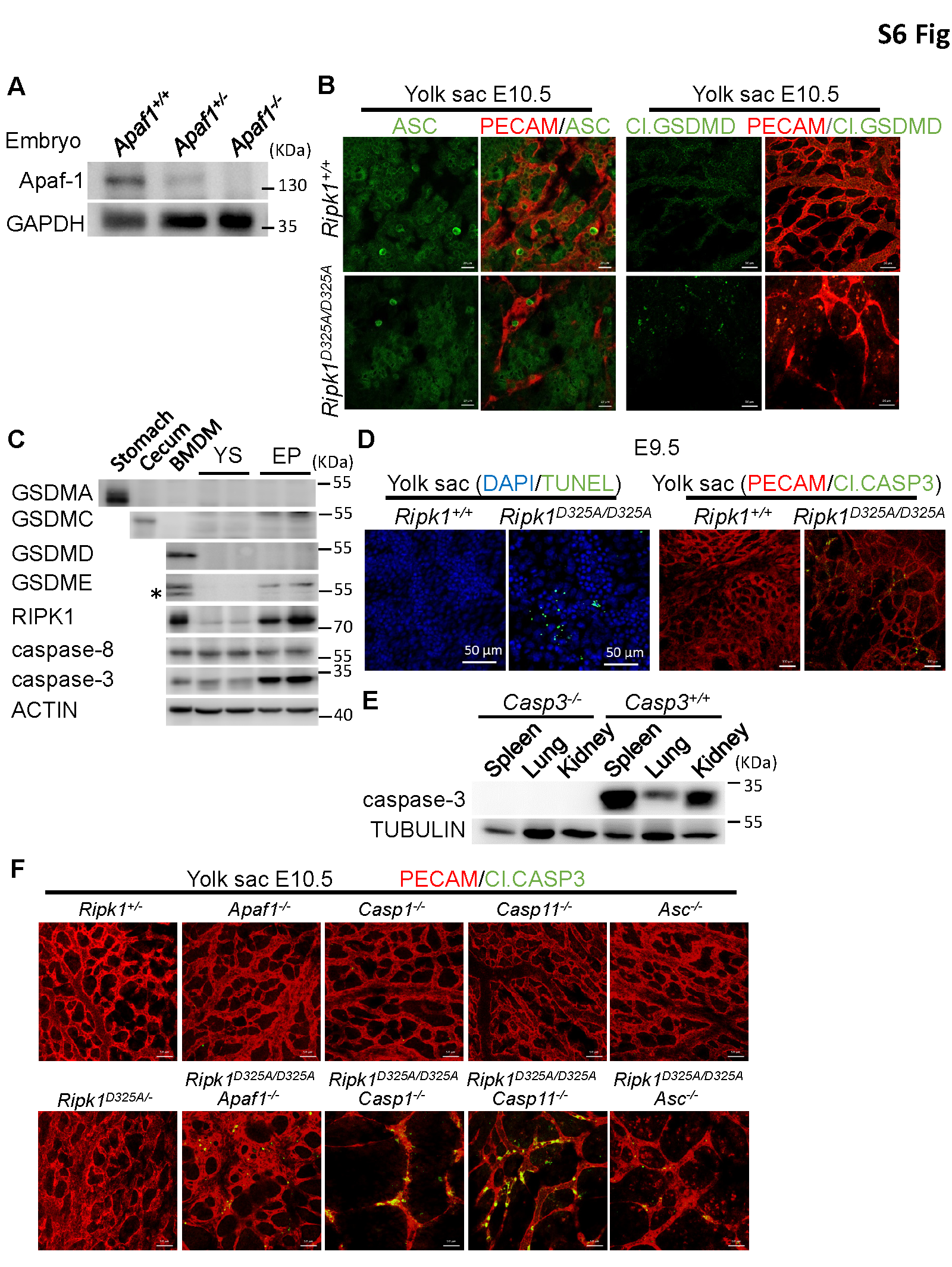

Supplement: S6 Fig — (A) Western blot analysis for Apaf-1 expression in embryos of indicated genotypes. (B) IF staining of E10.5 YS of indicated genotypes with anti-PECAM (red) and anti-ASC (green) or anti-Cl.GSDMD (green) antibodies. Scale bars, 50 μm. Images are representative of 3 embryos per genotype. (C) Western blot analysis for expression of gasdermins in E10.5 WT mouse embryos. Antibodies against the conserved region of 3 GSDMAs and conserved region of 4 GSDMCs, GSDMD, and GSDME were used. Anti-RIPK1, anti-caspase-8, and anti-caspase-3 antibodies were included as controls. *: nonspecific band. Panels A–C are related to Fig 6. (D) TUNEL assay of E9.5 YS and IF staining of E9.5 YS with anti-PECAM (red) and anti-Cl.CASP3 (green) antibodies. DAPI, a DNA stain. Scale bars, 50 μm. Images are representative of 3 embryos per genotype. (E) Western blot analysis for caspase-3 expression in different tissues from mice of indicated genotypes. (F) IF staining of E10.5 YS of indicated genotypes with anti-PECAM (red) and anti-Cl.CASP3 (green) antibodies. Scale bars, 50 μm. Images are representative of 3 embryos per genotype. Panels D–F are related to Fig 7. Uncropped immunoblot for panels A, C, and E can be found in S1 Raw Images. Apaf-1, apoptotic protease activating factor 1; ASC, apoptosis-associated speck-like protein containing a CARD; BMDM, bone marrow–derived macrophage; Cl.CASP3, cleaved caspase-3; Cl.GSDMD, cleaved GSDMD; E10.5, embryonic day 10.5; EP, embryo proper; GAPDH, glyceraldehyde 3-phosphate dehydrogenase; IF, immunofluorescence; PECAM, platelet endothelial cell adhesion molecule; RIPK1, receptor interacting serine/threonine kinase 1; TUNEL, terminal deoxynucleotidyltransferase-mediated dUTP-biotin nick end labeling; WT, wild-type; YS, yolk sac. (TIF) [file pbio.3001304.s006.tif]

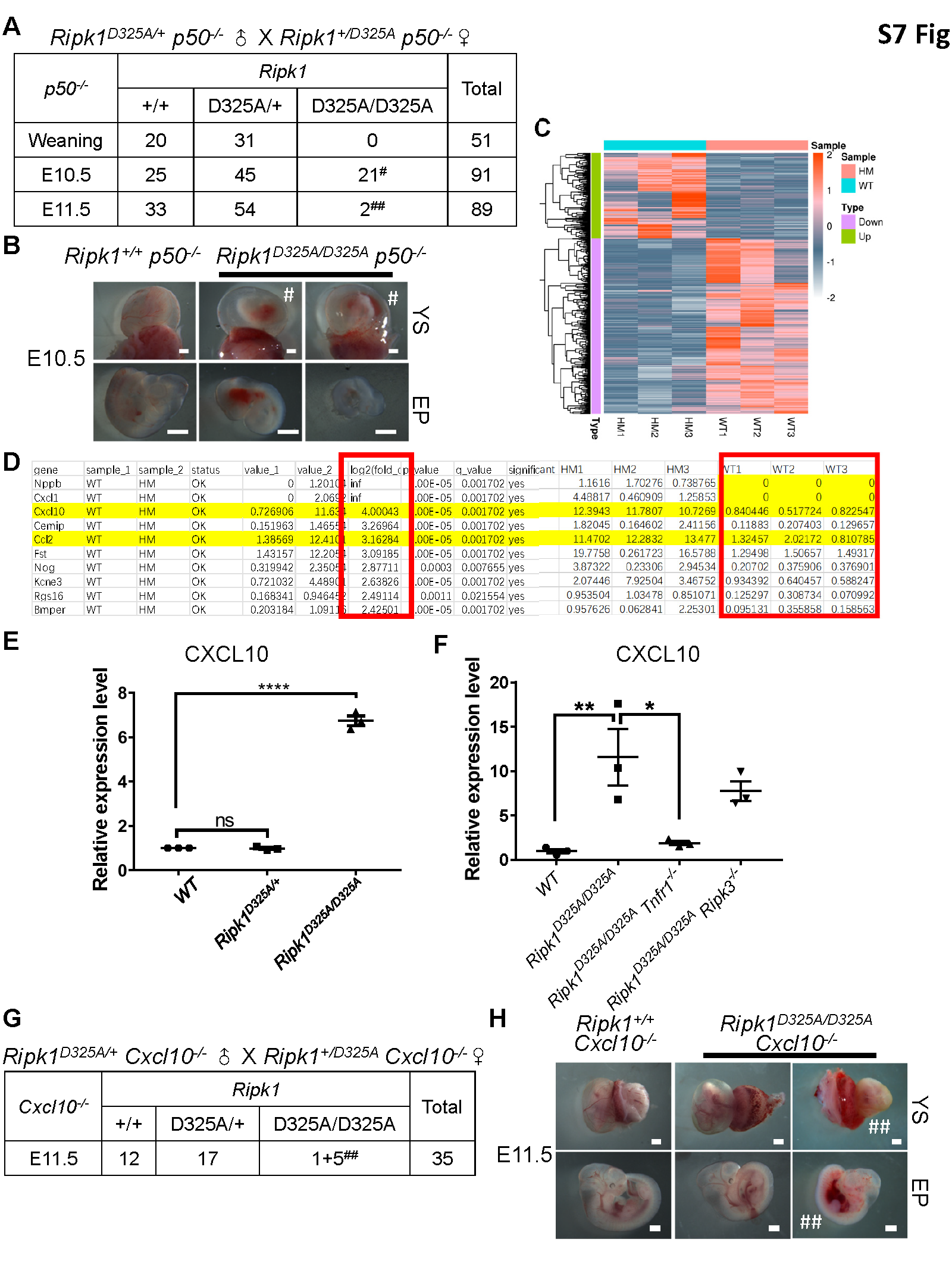

Supplement: S7 Fig — (A) Genetic analysis of offspring from intercrosses of Ripk1D325A/+ p50−/− parents. (B) Representative E10.5 embryos collected in (A). Scale bars, 1 mm. (C) RNA sequencing of YS RNA from WT and Ripk1D325A/D325A mice, both with biological triplicates. Heat map shows differentially expressed genes. (D) A list of top 10 high expression genes along with their parameters and values analyzed in (C). (E and F) Quantitative PCR analysis of CXCL10 expression in E10.5 YS. Data from 3 independent experiments are analyzed, 3 embryos per genotype per experiment. Data are represented as mean ± SEM. ns, no significance; ****: p < 0.0001; **: p < 0.01; *: p < 0.05. (G) Genetic analysis of progeny from intercrossing Ripk1D325A/+ Cxcl10−/− mice. (H) Representative images of E11.5 embryos obtained in (G). Scale bars, 1 mm. Underlying data are available in S1 Data. E10.5, embryonic day 10.5; E11.5, embryonic day 11.5; EP, embryo proper; HM Ripk1D325A/D325A homozygotes; NF-κB, nuclear factor-kappa B; WT, wild-type; YS, yolk sac. (TIF) [file pbio.3001304.s007.tif]
