# Supplementary material for: A unique death pathway keeps RIPK1 D325A mutant mice in check at embryonic day 10.5
Source: PLoS Biol. 2021 Aug 26;19(8):e3001304. doi: 10.1371/journal.pbio.3001304 (PMC8389420; doi:10.1371/journal.pbio.3001304)
Supplement: S1 Raw Images — (PDF) [file pbio.3001304.s009.pdf]

S1A Fig, left panel

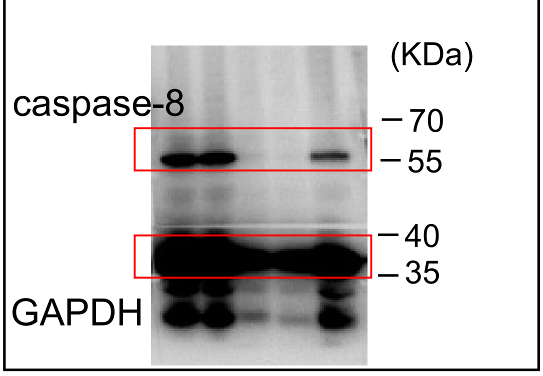

S1A Fig, right panel & S1D Fig

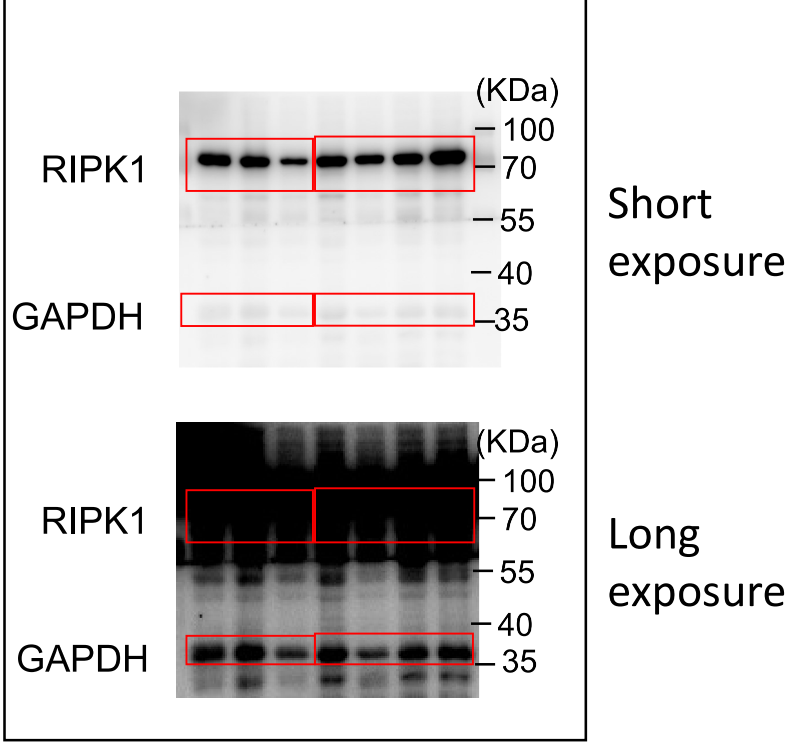

S5A Fig, middle panel

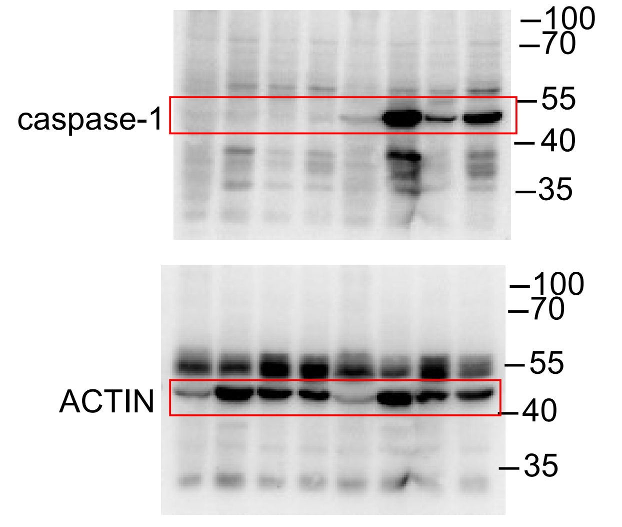

S5A Fig, left panel

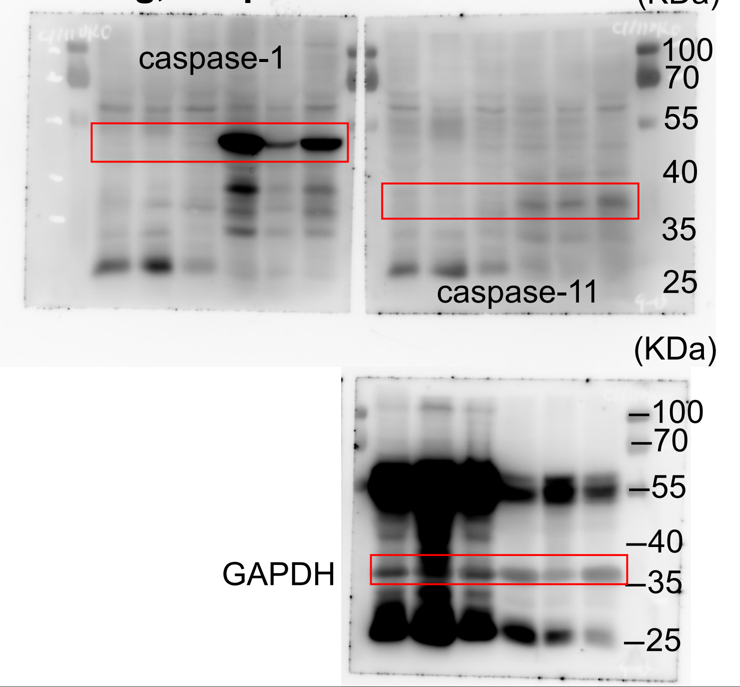

Antibody information
mouse anti-GAPDH (Abclonal, AC002)
mouse anti-RIPK1 (BD Biosciences, 610459)
rabbit anti-caspase-8 (Cell Signaling Technology, 4790S)
mouse anti-ACTIN (Cell Signaling Technology, 3700T)
rat anti-caspase-1 (Genentech, a gift from Vishva M Dixit)
rat anti-caspase-11 (Cell Signaling Technology, 14340S)

S5A Fig, right panel

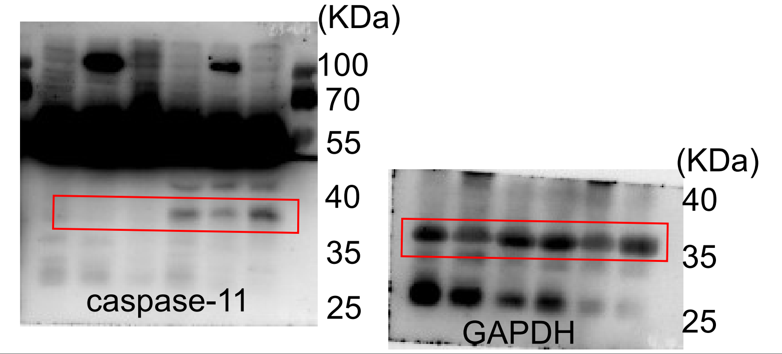

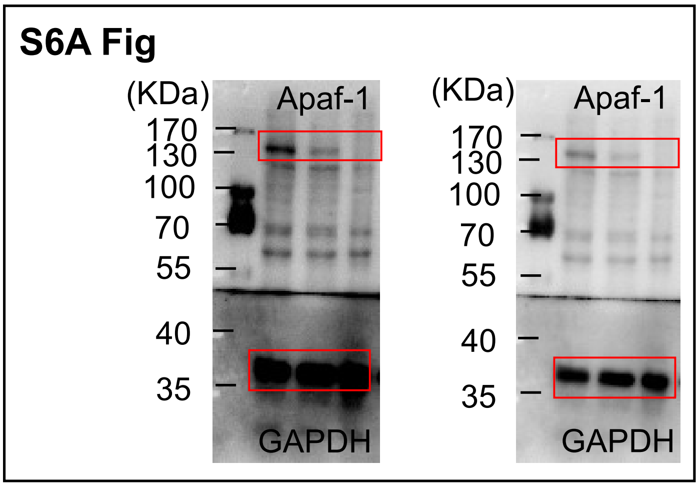

Long exposure Short exposure

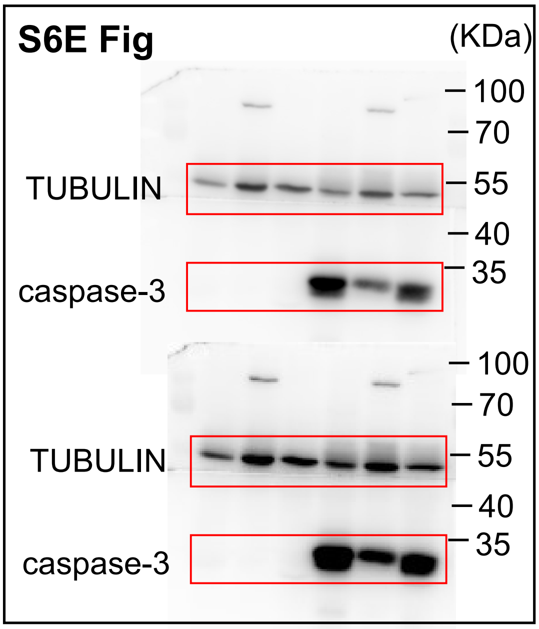

Short exposure

Long exposure

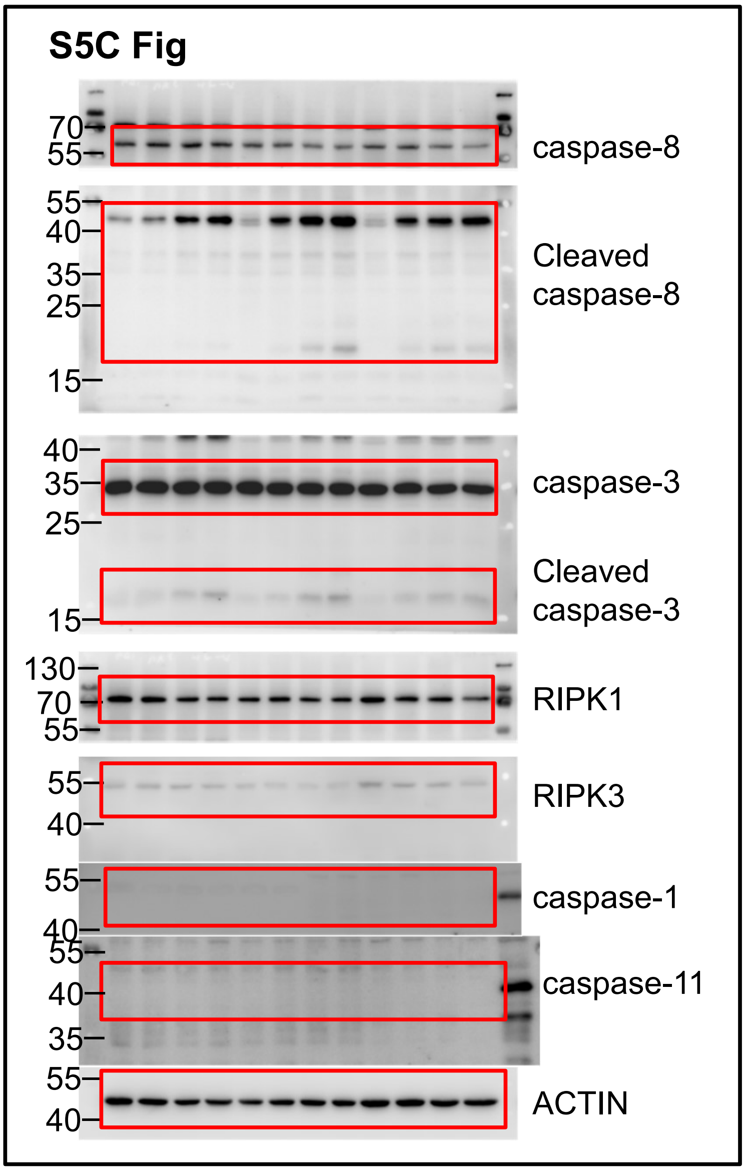

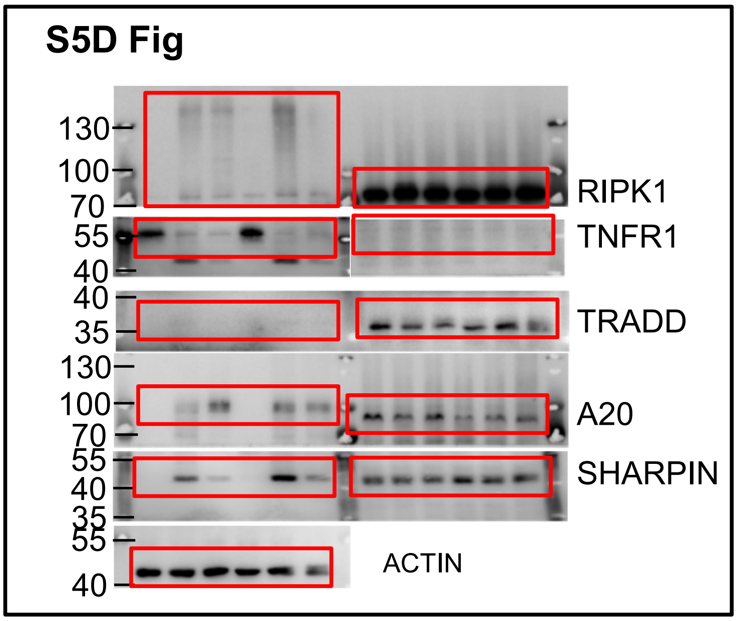

Antibody information
mouse anti-GAPDH (Abclonal, AC002)
mouse anti-TUBULIN (Abmart, M20005M)
rabbit anti-caspase-3 (Cell Signaling Technology, 9662S)
rabbit anti-Apaf-1 (Cell Signaling Technology, 8723S)
rabbit anti-caspase-8 (Cell Signaling Technology, 4790);
rabbit anti-RIPK1 (Cell Signaling Technology, 3493s)
Rabbit anti-RIPK3 (Cell Signaling Technology, 13526)
rabbit anti-Cleaved-caspase-8 (Cell Signaling Technology 9429)
mouse anti-ACTIN (Santa Cruz Biotechnology, AB_626632)
rat anti-caspase-1 (clone 4B4, a kind gift from Dr. Vishva M. Dixit, Genetech, USA)
rat anti-Caspase-11 (Cell Signaling Technology, 14340S)
rabbit anti-TNFR1 (proteintech, 21514-1-AP)
rabbit anti-TRADD (abcam, ab110644-100ul)
rabbit anti-A20 (Cell Signaling Technology, 5630S)
rabbit anti-SHARPIN (proteintech,14626-1-AP)

S5E Fig

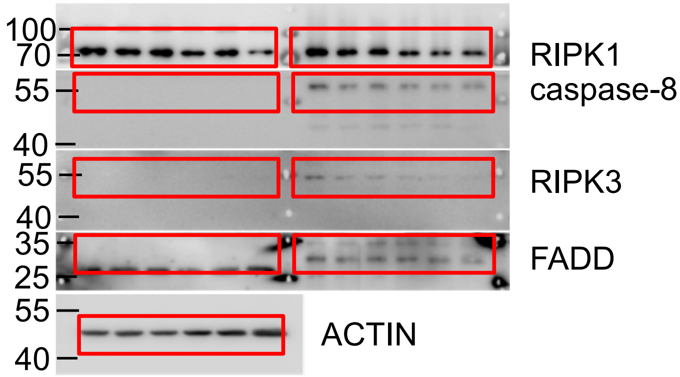

S6C Fig

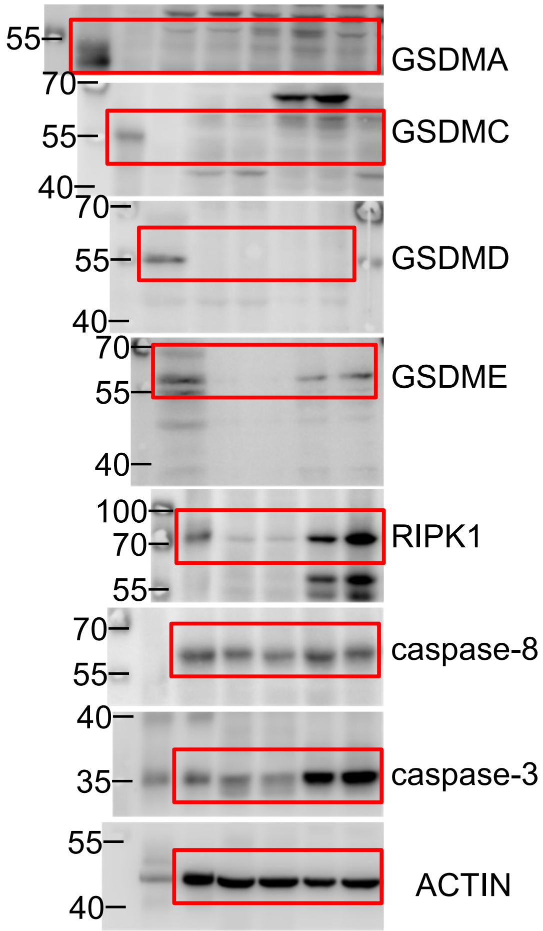

S5F Fig

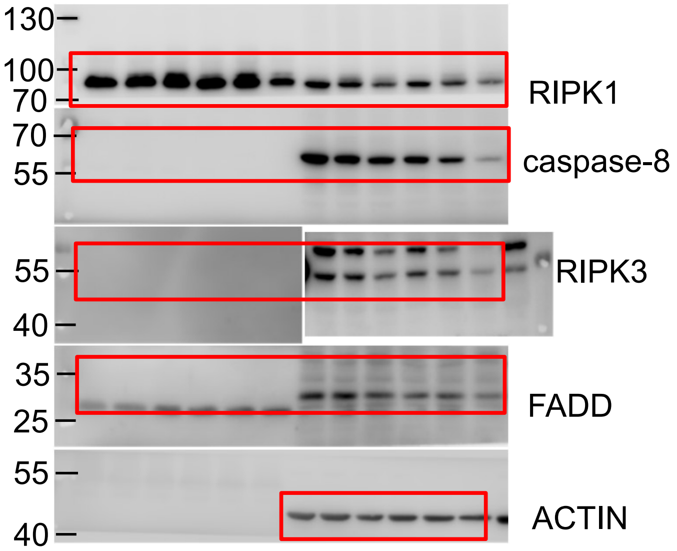

S5G Fig

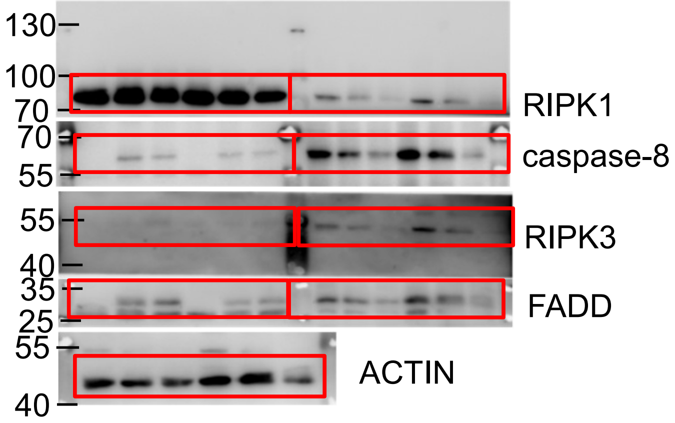

Antibody information
rabbit anti-caspase-8 (Cell Signaling Technology, 4790);
rabbit anti-RIPK1 (Cell Signaling Technology, 3493s)
Rabbit anti-RIPK3 (Cell Signaling Technology, 13526)
rabbit anti-caspase-3 (Cell Signaling Technology, 9662S)
mouse anti-ACTIN (Santa Cruz Biotechnology, AB_626632)
rabbit anti-FADD (proteintech, 14906-1-AP)
rabbit anti-GSDMD (abcam, ab209845)
rabbit anti-GSDME (abcam, ab215191)
rabbit anti-GSDMA (immunogen: GST-GSDMA (248-347aa), homemade)
rabbit anti-GSDMC (immunogen: GST-GSDMC2 (367-467aa), homemade)
